# Comparing resting state and task-based EEG using machine learning to predict vulnerability to depression in a non-clinical population

**DOI:** 10.1038/s41598-023-34298-2

**Published:** 2023-05-08

**Authors:** Pallavi Kaushik, Hang Yang, Partha Pratim Roy, Marieke van Vugt

**Affiliations:** 1 Bernoulli Institute of Mathematics, Computer Science and Artificial Intelligence, University of Groningen, Nijenborgh 9, 9747 AG Groningen, The Netherlands; 2 Department of Computer Science and Engineering, Indian Institute of Technology Roorkee, Roorkee, 247667 India

**Keywords:** Biomedical engineering, Computational science, Quality of life, Psychology, Human behaviour

## Abstract

Major Depressive Disorder (MDD) affects a large portion of the population and levies a huge societal burden. It has serious consequences like decreased productivity and reduced quality of life, hence there is considerable interest in understanding and predicting it. As it is a mental disorder, neural measures like EEG are used to study and understand its underlying mechanisms. However most of these studies have either explored resting state EEG (rs-EEG) data or task-based EEG data but not both, we seek to compare their respective efficacy. We work with data from non-clinically depressed individuals who score higher and lower on the depression scale and hence are more and less vulnerable to depression, respectively. Forty participants volunteered for the study. Questionnaires and EEG data were collected from participants. We found that people who are more vulnerable to depression had on average increased EEG amplitude in the left frontal channel, and decreased amplitude in the right frontal and occipital channels for raw data (rs-EEG). Task-based EEG data from a sustained attention to response task used to measure spontaneous thinking, an increased EEG amplitude in the central part of the brain for individuals with low vulnerability and an increased EEG amplitude in right temporal, occipital and parietal regions in individuals more vulnerable to depression were found. In an attempt to predict vulnerability (high/low) to depression, we found that a Long Short Term Memory model gave the maximum accuracy of 91.42% in delta wave for task-based data whereas 1D-Convolution neural network gave the maximum accuracy of 98.06% corresponding to raw rs-EEG data. Hence if one has to look at the primary question of which data will be good for predicting vulnerability to depression, rs-EEG seems to be better than task-based EEG data. However, if mechanisms driving depression like rumination or stickiness are to be understood, task-based data may be more effective. Furthermore, as there is no consensus as to which biomarker of rs-EEG is more effective in the detection of MDD, we also experimented with evolutionary algorithms to find the most informative subset of these biomarkers. Higuchi fractal dimension, phase lag index, correlation and coherence features were also found to be the most important features for predicting vulnerability to depression using rs-EEG. These findings bring up new possibilities for EEG-based machine/deep learning diagnostics in the future.

## Introduction

Major Depressive Disorder (MDD) is one of the most prevalent mood disorders, affecting more than 264 million people worldwide^1^. It has become so common that 1 in 15 adults is affected by it in a given year. Depression affects the way one feels, thinks and acts. Its core symptoms include sadness and feelings of anhedonia, i.e. loss of interest in activities one once enjoyed, leading to decreased task performance and productivity. In its extreme form, it can even lead to suicide which is the fourth leading cause of death in 15–29 year-olds and according to the World Health Organization, close to 700,000 people die due to suicide every year^2^. Another major concern pertaining to depression is that even after it has been diagnosed and cured, there is still a high chance of relapse in the patients. There is no effective mechanism to track depression and predict relapse yet, and owing to the social stigma associated with depression, it often goes undetected and untreated. In recent years, an increased emphasis on mental health and access to care via telehealth has increased the number of patients seeking treatment. However, cost-effectiveness models suggest that even in the unlikely event of optimal treatment being delivered in all cases, only 35–50% of the overall burden of depression and anxiety would be alleviated^3^. Thus, there is a dire need to find a sensitive biomarker that spans the continuum from health to disease so that even minor deviations can be tracked and relapse be detected for effective early intervention.

Rumination has been found to be strongly and most consistently related to depressive symptoms^4^. According to Response Styles Theory^5^, rumination is characterised by excessive focus on self^6^ as well as a repetitive and passive focus on one’s negative emotions^7^. Rumination has important implications in the manifestation, understanding and maintenance of depressive episodes and has been shown to prolong and deepen episodes of depression by persevering in a depressed mood^8^. It has also been found to most consistently predict depression and onset of depression^4^, making rumination the key biomarker of interest for understanding relapse. Emotion regulation and reward processing^9,10^ are other paradigms apart from rumination that have been used in depression studies. However, we focus on rumination in this study as it is the most noteworthy symptom an affected person and the people around them experience.

Rumination can be evoked in a laboratory setting, for example, in a Sustained Attention to Response Task (SART), especially when it is preceded by a validated social stressor^11^, such as the Trier Social Stress Task^12^ or when participants are asked about their failures before starting with the task. These studies have found that depressed individuals report more instances of rumination (being off-task). Moreover, an interesting aspect of negative rumination that has received attention recently is the “stickiness” of thoughts: the difficulty in disengaging from the thoughts causes prolonged and repeated pondering on thoughts. This cognitive variable can be a reliable factor in studying the non-clinical manifestation of rumination^13^.

Vugt and Broers^14^ measured “sticky” thoughts in a SART and found that they can be identified by including “thought probes” in the SART, by asking the participant what they are thinking about at that moment. They reported that “stickiness” had substantial effects on the performance of the participants in the task and indicated that an increase in self-reported “stickiness” instances may be related to tendencies of depression. Level of “stickiness” has also been shown to be associated with pupil size^15^. In this study, we explore if EEG signals can capture this subtle state and allow more reliable differentiation, as pupil size is sensitive to arousal in general, whereas EEG has been related to a variety of functions, including attention, memory and emotion. Additionally, a unidimensional measure like pupil size might be a good choice when one knows what parameters affect the data and what one is precisely looking for but when we don’t, it’s helpful to have as much information as possible and multidimensional signals are a more plausible choice.

EEG signals are multidimensional signals with a low signal-to-noise ratio. Data-driven tools like machine learning have been used to find relevant patterns within groups of data that are not possible for humans to comprehend. Since we are interested in relapse, we work with data from otherwise healthy people who score higher on various questionnaires related to depression and its symptoms and hence are more or less vulnerable to the disorder rather than clinically depressed individuals. This study explores if significant statistical differences lie between EEG signals of individuals in these two groups (high vs low vulnerability to depression) and if machine/deep learning can help differentiate them using EEG data corresponding to the “stickiness” of thoughts.

A lot of studies have also explored the prediction of whether an individual suffers from MDD using resting-state EEG (rs-EEG) data and machine/deep learning^16–18^. These studies tried to discover depressogenic markers exploiting evoked potentials, band power, functional connectivity, linear signal features (e.g., relative gamma power, alpha power variation) and non-linear (Higuchi Fractal Dimension (HFD), Detrended Fluctuation Analysis (DFA)) signal features^19^. Hosseinifard et al.^17^ report a classification accuracy of 90% by combining all nonlinear features (HFD, correlation dimension and Lyapunov exponent) using logistic regression classifier. Khan et al.^20^ report achieving an accuracy of 98.1% using a 2d-convolution neural network using coherence as a feature. However, there is no consensus as to which feature can be generalized across all/most patients. For example, Hosseinifard et al.^17^ found that alpha and theta bands, especially in the left hemisphere, are good features to differentiate depressed from healthy controls, whereas Shen et al.^21^ found that alpha-based features had less prediction power than beta, theta and gamma based features while differentiating the two groups. In this study, we explore if already established biomarkers of MDD are also capable of differentiating between healthy and depression-prone individuals. We also use evolutionary algorithms, namely, Grey Wolf Optimization (GWO)^22^, Genetic Algorithm (GA)^23^ and Particle Swarm Optimization (PSO)^24^ to determine a subset of these vast number of biomarkers that are sufficient to differentiate these two groups.

As far as we searched, SART has not been used to predict depression using machine/deep learning. However, other tasks like response and approach to reward learning and emotion regulation for predicting depression using machine learning have been used. Kean et al.^9^ employed eye tracking, behavioural questionnaires, and EEG data to model an elastic net to predict the most accurate predictors of self-related reward processes utilising numerous tasks related to attention, processing, and memory for valued information. The model anticipated variance in the test set in the approach motivation and response responsitivity to be between 5–12% and 9.7–28%respectively. Leticia et al.^10^ collected fMRI data while participants viewed happy, sad and neutral faces. Gaussian process classifier was used to compare the predictive probabilities of brain activation patterns to emotional and neutral faces between healthy controls and depressed patients. The predictive probabilities to neutral faces in patients were 0.65 compared to 0.75 in healthy controls.

**Figure 1:**
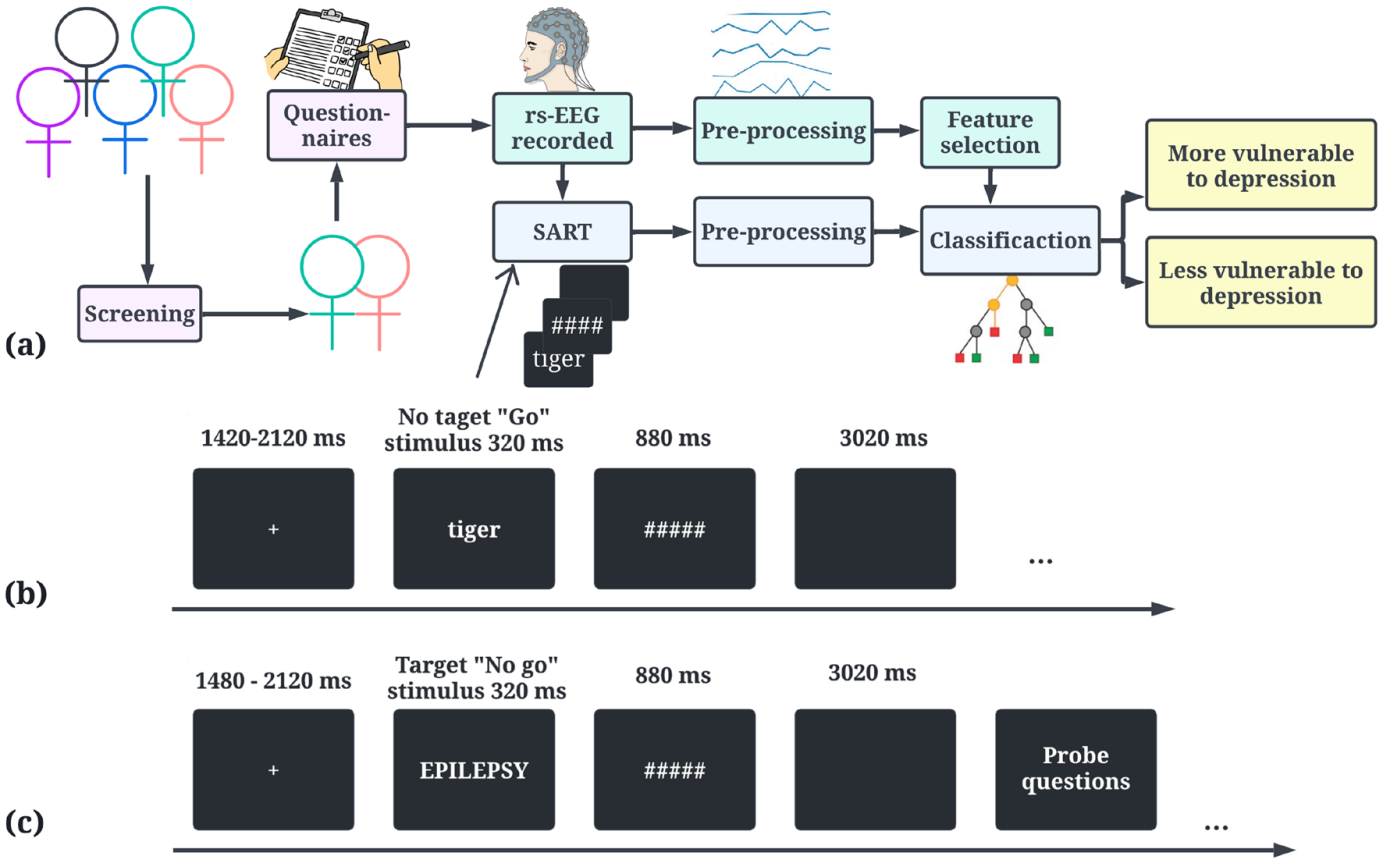
(**a**) Framework of the recognition system used for differentiating vulnerability (more/less) to depression. (**b**) a SART task showcasing the different components of a trial without probe questions. (**c**) a SART task showcasing the different components of a trial with probe questions.

Most of the studies either used rs-EEG or task-based EEG, but not both from the same participants to predict MDD status. Hence, we do not know their predictive capabilities, and in this study, we work with both data to get a broad understanding of the respective efficacy of each method. The objective of the above-mentioned analyses is to inform us whether the rs-EEG that is easier to collect but is entirely agnostic of the actual thinking process a depressed individual goes through, is more informative and effective in differentiating between people with high and low vulnerability to depression than a task-based method (SART). The framework of the study is shown in Fig. 1a.

## Materials and methods

### Dataset description

#### Pre-EEG questionnaires

To explore the vulnerability to perseverative cognition and depression, participants were invited to fill in several online questionnaires: the Perseverative Thinking Questionnaire (PTQ) measuring repetitive negative thinking^25^, Rumination Response Scale (RRS) measuring depressive rumination^26^ and the Center for Epidemiologic Studies Depression Scale (CES-D) for the severity of depression^27^. The score of each questionnaire was standardised (z-score) separately and summed up, and the total score was obtained for each participant. Based on the total scores, the top and bottom 20 participants were further selected for each group and they were within the distribution of the top 23.53 % or bottom 30.39 % respectively of the total score.

After the screening, the selected participants were further asked to fill in a number of questionnaires: (1) Cognitive Failures Questionnaire-Memory and Attention Lapses (CFQ-MAL)^28,29^; (2) Personal Concerns Inventory (PCI)^29^; (3) Adult ADHD Self-Report Scale^29^ and (4) Action Orientation Scale (AOS)^30^. The PCI was designed to access participants’ most important concerns and worries via subjective rating. The content of the concerns and worries were transformed into word triplets, which were further inserted into the SART to provoke worrisome and depressive episodes without awareness^29^. For example, if the participant had been supported by his/her family and reported being worrisome about finding a job, the information was converted into a sequence of “financial-support-employment” embedded in the SART. The other three questionnaires played the role of filler questionnaires.

To further increase the occurrence of ruminative thinking, participants were asked to nominate a negative event in life and write a description of the event in ten minutes preceding the EEG recording. The writing task was designed to further designed to provoke negative episodes and thus increase the occurrence of depressive rumination. A 6-point Likert Score was added after the writing, asking how strongly the event affect them and how often it bothered the participant^31^ Due to incomplete filling in of the writing manipulation, we are missing three data points in the high vulnerability group and one data point in the low vulnerability group in the writing manipulation.

#### Participants

Among the 102 participants who completed the questionnaires on perseverative cognition and depression, forty of them were recruited into the high vulnerability group (20 in total, 16 female and 4 male) and low vulnerability group (20 in total, 9 female and 11 male) based on the total scores of pre-EEG questionnaires (including PTQ, RRS and CES-D). The two groups were balanced in their handiness according to the chi-square test ($\chi ^2$ = 1.03, df = 1, *p* = 0.31, $BF_{10}$ = 1.03). There was a group difference in the proportion of gender ($\chi ^2$ = 5.23, df = 1, *p* = 0.02, $BF_{10}$ = 4.87) which was expected due to the higher prevalence of depression among the female population^32^. The Bayes Factor (BF) was calculated with the contingencyTableBF function of the BayesFactor package^33^. The effect of gender was not controlled in the further statistics. The participants were recruited through the social media of the university. Their age was not asked throughout the experiment in accordance with GDPR regulations that prevent us from collecting information that is not absolutely critical for the study. Since the effects of gender were not of interest to the main research question, we did not pursue this further. All participants were confirmed to have a normal or corrected-to-normal vision before the experiment. A fee of 8 euro/h was paid for their participation.

#### Ethics approval and consents

All procedures performed in studies involving human participants were in accordance with the ethical standards of the institutional and/or national research committee and with the 1964 Helsinki declaration and its later amendments or comparable ethical standards. The study protocol was approved by the CETO (Research Ethics Review Committee of the Faculty of Arts of the University of Groningen). All the participants gave informed consent which was approved by the CETO.

#### EEG recording

EEG signals were recorded using a Biosemi system (Biosemi, Amsterdam, Netherlands) with a sampling rate of 512 Hz. The system includes 32 electrodes on the cap (as shown in Fig. 1a) and 6 loose electrodes including two near the mastoid and four measuring eye movements. The configuration of the channels followed the layout of 10–20 system^34^. The EEG recording used the Cz electrode as the online reference. For every participant, the impedance was kept below 20 k$\Omega$ during the experiment.

#### Resting state EEG

After setting up the EEG equipment, participants were told to start a resting state period which lasted for 5 minutes following the instruction of an existing study^35^. Participants were asked to sit quietly and be relaxed, close their eyes while trying not to sleep. A sound which followed a button press marked the start of the resting state and participants were told to keep their eyes closed until another sound beep when the five minutes ended.

#### SART procedure

As was typically used in existing studies on task-unrelated thought^36,37^, the SART was used to track ruminative thinking in this study. The SART was a simple task with ample time to mind-wander , which was following the paradigm of the existing study^29^. In the SART procedure, a trial consisted of a cross (“+”) for an average of 1800 *ms* in the very beginning, a word for 320 ms and a mask for 880 ms to guide the responses. After the mask, an Inter-Stimulus Interval (ISI) for 3020 ms was followed. The duration of the cross was sampled from 1480, 1640, 1800, 1960, 2020 ms to prevent anticipation before the word appeared. A number of 720 word items derived from^38^ were pseudo-randomised after inserting word triplets extracted from the PCI (30 triplets in the whole experiment)^29^. The task of the participant was to respond with the “N” key every time a lowercase word (“Go” stimulus, made up to 8/9 of all the trials) appeared and withhold the response when an uppercase word (“No-go stimulus”, made up to 1/9 of the trials). There were eight blocks in total for the SART, and each block consists of ninety trials.

Four types of thought probes were inserted into the SART asking participants consecutively the content of the thoughts, the degree of self-focus, the valence of the thoughts and the stickiness of the thoughts. The entire study design and probe questions are shown in Fig. 1b,c and Supplementary Fig. 1 respectively. Participants had to indicate a number from 1 to 6 (for the content probe) or from 1 to 9 (for the other three thought probes). To find if “stickiness” induced by perseverative thinking in rumination is predictive of depression, we will be focused on the stickiness dimension of the thoughts in this study. The five trials before the thought probes were marked with their stickiness. Based on responses to the 9-point Likert Scale, we further classified these trials ranging from 1 to 4 as “less sticky”, and the trials from 6 to 9 as “more sticky”. The EEG data corresponding to these five trials before each probe was combined together to form the dataset for stickiness. In this study, we do not look into the differences between high and low sticky data but rather combine these data under the umbrella of sticky data and use it to differentiate high vs low vulnerability to depression groups. “Less sticky” and “more sticky” data combined are termed task-based EEG data in the paper henceforth.

All the stimuli except the blank screen was kept flickering on and off at a frequency of 12.5 Hz in an effort to evoke the Steady-State-Visual-Evoked-Potentials (SSVEPs) which will not be further discussed in this paper but it was reported in Yang et al.^39^. The procedure was designed with self-written codes in Psychopy in the environment of Python^40^. A refresh rate of 50 Hz was set for the screen which was a 65 cm distance away from the participants. The visual angle of the word stimuli ranged from $5.72\,^\circ$ to $36\,^\circ$ horizontally and remained at $5.5\,^\circ$ vertically.

**Table 1:** The average and standard deviation (in brackets) of the questionnaire scores and ratings of the negative events across the high vulnerability and low vulnerability to depression groups.

Group	PTQ scores	RRS scores	CES-D scores	Intensity	Frequency
High vulnerability to depression	45.85 (4.70)	67.05 (7.34)	51.65 (6.47)	4.76 (0.97)	4.35 (0.86)
Low vulnerability to depression	25.10 (8.33)	41.75 (8.34)	35.45 (3.71)	4.11 (0.74)	3.84 (0.83)
Difference between groups	t(38) = 9.70, *p* = $7.87 \times 10^{-12}$	t(38) = 10.19, *p* = $2.05 \times 10^{-12}$	t(38) = 9.71, *p* = $7.66 \times 10^{-12}$	t(34) = 2.31, *p* = 0.027	t(34) = 1.81, *p* = 0.08

#### EEG analysis

*EEG pre-processing*. The continuous EEG data of the SART were firstly filtered with a band-pass filter (0.5–40 Hz) in EEGLAB^41^ and MATLAB (The Mathworks, Inc.), and then segmented into epochs starting from 500 ms before stimulus onset to 1300 ms after. The 1300 ms after stimulus onset was selected to make sure the segmentation covers the time course of the related cognitive processes. The time interval before stimulus onset was used for baseline correction. By visually inspecting the EEG across all the trials, trials with artefacts caused by slow wave drifts, and abrupt head or muscle movement were manually marked and rejected for the following analysis. A proportion of 12.72 % (varied from 2.14 to 43.59 % across individuals) of the trials was removed in the pre-processing stage. The channel signal was interpolated with the average of neighbouring channels if bad channels were found according to the notes during data recording or visual inspection during the offline data analysis. Bad channels were found among two participants (one for each participant). An Independent Component Analysis (ICA) was implemented in order to remove artefacts which are related to eye blinks, saccades and muscle activities following the re-referencing based on the average of all non-EOG electrodes. Independent components which were related to eye blinks, saccades and muscle activity were removed from the signal (a maximum of 5 components were removed). In order to select a shorter time period as the baseline, the epochs ranging from − 200 to 1300 ms after stimulus onset were selected for further analysis. A proportion of 12.72 % (varied from 2.14 to 43.59 % across individuals) of the trials were removed in the pre-processing stage.

### Correction for multiple comparisons

An issue with EEG studies is that there are many electrodes and when statistical tests are done on each individual electrode for each brain wave, that leads to an inflation of the false positive rate. To correct this, we used the False Discovery Rate (FDR)^42^. A false discovery threshold level of 0.05 was used for all the analyses reported henceforth. Details on feature extraction are in Supplemental Methods.

### Classification using machine/deep learning

It is a standard practice in machine/deep learning to compare the classification results from various classifiers to ensure that the results are generalizable and not governed by any bias of a single classifier. We experimented with various classifiers, MultiLayer Perceptron (MLP)^43^, Decision Tree (DT)^44^, 1D-Convolution Neural Network (CNN)^45^, Long Short Term Memory (LSTM)^46^ and Bidirectional LSTM (BLSTM). Both the rs-EEG and task-based datasets are fairly balanced with rs-EEG having 4,702,208 samples for less vulnerable and 4,609,536 samples for more vulnerable individuals. The task-based dataset comprises 2,717,481 and 2,185,183 samples for less and more vulnerable groups’ respectively. Here, ’samples’ mean the EEG data points collected from participants over time-so each sample is an individual data point, and it is a vector with a dimension of 32 because of the 32 EEG channels used in data collection.

The EEG data collected from the participants were used to train the models. The data corresponding to all participants who had a low vulnerability to depression were combined together under one label while the data from all individuals with high vulnerability to depression were combined together under another label. A dataset was prepared like this for rs-EEG, and task-based EEG, respectively. Machine/deep learning models were trained on these datasets separately to determine whether an EEG sample belonged to a person highly vulnerable to MDD or low vulnerable to MDD. For machine learning algorithms (MLP and DT) k-cross validation was used with k = 5 giving the maximum accuracy. Since hyperparameter tuning is required for deep learning (CNN LSTM, and BLSTM), data were split into training, validation and test sets in the ratio of 60:20:20 respectively. We experimented with various models, trying different permutations of layers, regularizers, optimizers, batch size, window size and activation functions (more details in Supplement methods: Hyperparameter tuning). The models that performed the best are reported in the manuscript. We used accuracy as the metric to evaluate the performance of the classifiers along with checking the confusion matrices for precision, recall and F1 score.

## Results

In this section, we first examine if individuals with high vulnerability to depression differ from individuals with low vulnerability to depression in terms of their questionnaire scores. We next explore which electrodes and features show statistically significant differences between these two groups on average both in the resting state and task-based (SART) EEG data. We also investigate whether it is possible to make use of these data to predict if an individual is more or less prone to depression in real-time on a single-trial level using machine/deep learning. As many biomarkers have been proposed for the rs-EEG in the literature, we then report which feature subset is more indicative of these differences in the rs-EEG using evolutionary algorithms.

### Statistical differences in the questionnaire scores

The group difference was examined with an independent t-test. The two groups showed a significant difference in questionnaire scores including PTQ, RRS and CES-D scores, as well as the rating of the intensity of the negative event (the detailed results are reported in Table 1).

**Figure 2:**
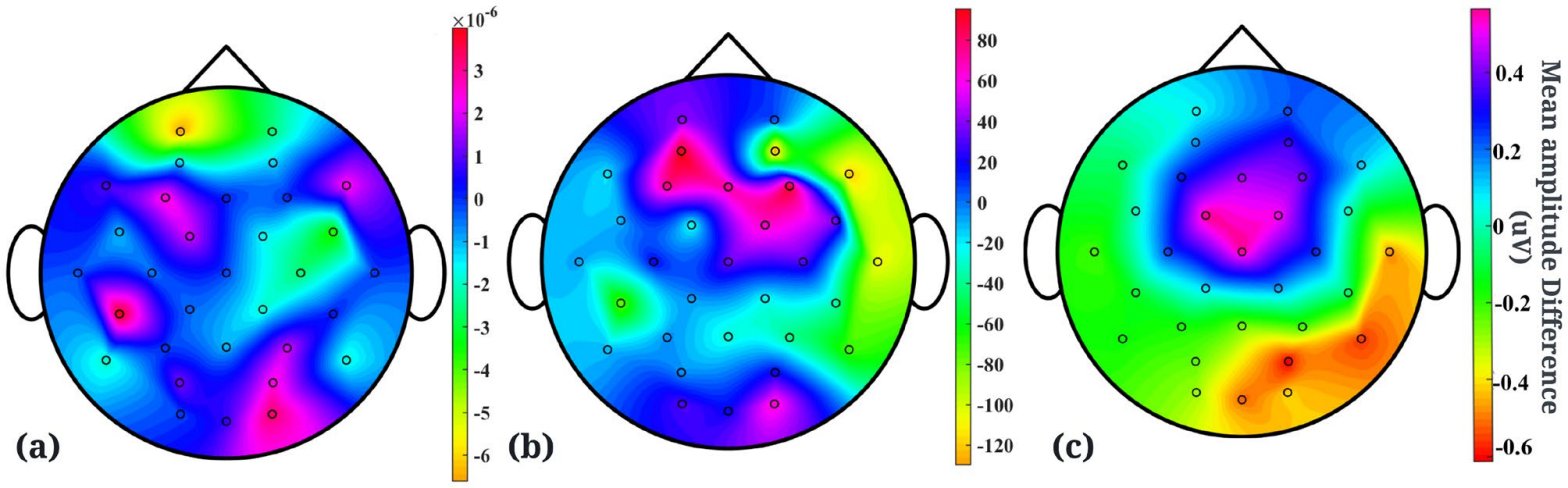
(**a**) Topographical map showing the difference in mean amplitudes (raw rs-EEG) between individuals with low and high vulnerability to depression. (**b**) Topographical map (rs-EEG delta) showing the difference in mean amplitudes between individuals with low and high vulnerability to depression. (**c**) Topographical map (delta) showing the difference in mean amplitudes between individuals with low and high vulnerability to depression for task-based data.

### Statistical differences between individuals with higher vs lower susceptibility to depression

#### Resting state EEG

**Figure 3:**
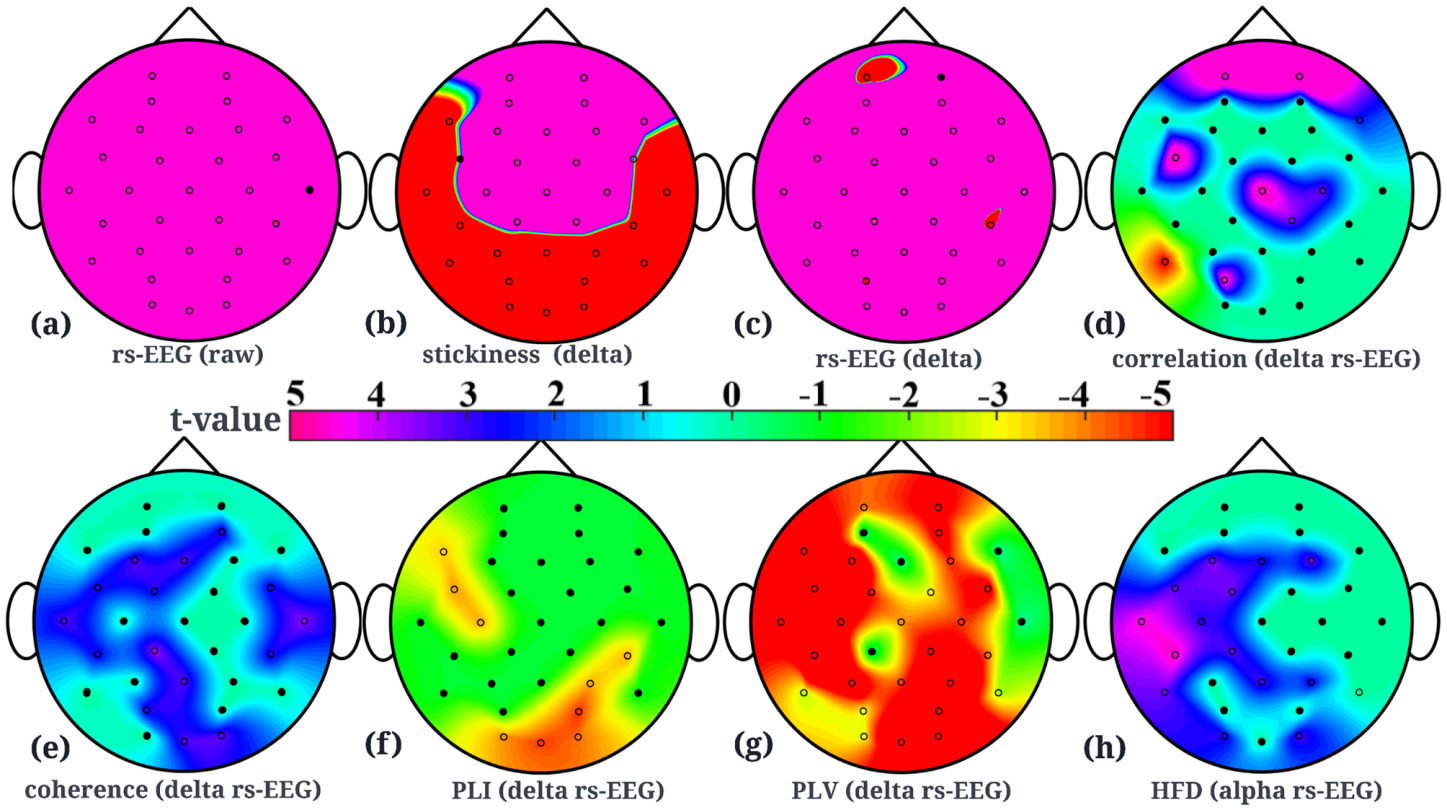
Topographical plots showing t-values of channels with significant *p*-values of difference between individuals with less and more vulnerability to depression. Channels with *p*-values above the threshold i.e. non-significant channels have been marked in black and corresponding t-values are plotted as zero for clarity. (**a**) Topographical plot for raw rs-EEG data with *p*-value threshold being 4.88e−17 after FDR. (**b**) Topographical plot of channels for task-based (stickiness) EEG data in delta wave with a *p*-value threshold of 0.0173 after FDR. (**c**) Topographical plot for delta brain wave (rs-EEG) with a *p*-value threshold of 0.1095e−11 after FDR. (**d**) Topographical plot of channels that show differences between the two groups in delta brain wave for correlation feature (*p*-value threshold 0.0142 after FDR). (**e**) Topographical plot for delta brain wave’s coherence feature (*p*-value threshold 0.0081 after FDR). (**f**) Topographical plot for delta brain wave’s Phase Lag Index (PLI) feature (*p*-value threshold 0.0141 after FDR). (**g**) Topographical plot for delta brain wave’s Phase Lock Value (PLV) feature (*p*-value threshold 0.0295 after FDR). (**h**) Topographical plot for alpha brain wave’s Higuchi Fractal Dimension (HFD) feature (*p*-value threshold 0.0066 after FDR). All the mentioned *p*-value thresholds are evaluated after multiple comparisons using FDR and correspond to a false discovery threshold level of 0.05.

Brain waves, alpha asymmetry, relative gamma power, functional connectivity measures (correlation, absolute value of coherence, imaginary part of coherence, phase lock value, phase lag index) and signal features like HFD, and DFA, are reported to be the most relevant depression biomarkers in recent studies^19^. In order to understand which (if any) of these biomarkers are representative of the differences between individuals with high and low vulnerability to depression, independent t-tests were conducted. Raw data (rs-EEG data after baseline correction and average re-referencing), had on average increased activity in the left frontal channel, and decreased activity in the right frontal and occipital channels for individuals more prone to depression than their counterparts as shown in Fig. 2a. t-value statistic (*p*-value threshold of 4.88e−17 corresponding to a false discovery threshold level of 0.05) showed that there is a widespread area of channels that show significant differences between the two groups as shown in Fig. 3a. *Depression biomarkers*: Amongst the brain waves, only the delta band showed significant differences (*p*-value threshold of 1.095e−11 corresponding to a false discovery threshold level of 0.05) in EEG activity as shown in Fig. 3c. Figure 2b shows that individuals more prone to depression were also seen to have on average increased activity in the frontal channels and decreased activity in the right occipital and parietal channels in the delta brain wave.

Mean Correlation and PLV across groups (more and less vulnerability to depression showed significant differences only in raw EEG data and lower frequency wave, delta. Figure 3d shows that correlation in delta brain wave has frontal channels that are statistically significantly different for the two groups. Figure 3g shows that mean PLV (delta band) across groups in the occipital, left temporal, parietal and frontal region are statistically significant in differentiating individuals with higher vulnerability to depression than those with lower vulnerability to the disorder. Mean coherence across participants in the two groups showed significantly different channels in delta (Fig. 3e) and gamma brain waves. Mean PLI across the two groups had significant channels in raw, delta and gamma brain waves. However, delta reported the most significant channels as shown in Fig. 3f, in the occipital region. Delta, theta and alpha waves showed significant differences in channels for HFD and Fig. 3h shows significant channels in the alpha wave.

No significant differences were found between the two groups for alpha asymmetry, relative gamma power, the imaginary part of coherence and DFA. Supplement Table I contains a detailed report of significant channels after FDR with corresponding topographical maps of all biomarkers for raw EEG data and brain waves.

#### Task-based EEG data

Another reasonable approach to differentiating individuals according to their vulnerability to depression is to use a cognitive task that taps into processes that are relevant to depression, most notably rumination. One candidate task for this is the SART task mentioned above that captures spontaneous thought, which should in principle be able to capture rumination, which is also a spontaneous thought process.

To examine if differences exist in task-based EEG data between individuals with higher and lower vulnerability to depression, we plotted the difference in mean amplitudes across individuals for different brain waves. To give an idea of activity in which channels differs between the two groups, Fig. 2c shows the differences in mean amplitude corresponding to the delta brain wave. It shows an increased activity in the central part of the brain for individuals with low vulnerability and an increased activity in the right temporal, occipital and parietal regions in individuals more vulnerable to depression. Refer to Supplement Table II for plots from all brain waves.

Independent t-tests followed by correction for multiple comparisons using FDR were also conducted using task-based EEG data. Only raw EEG and lower frequency bands, delta and theta yielded channels that are significant in distinguishing individuals with higher vulnerability from those with lower vulnerability to depression. Fig. 3b shows a widespread area of significant channels after correction for multiple comparisons (*p*-value threshold of 0.0173 corresponding to a false discovery threshold level of 0.05). Please refer to Supplement Table I for plots from all brain waves. We also conducted independent t-tests to explore if differences exist between the two groups based on their respective “high” stickiness and “low” stickiness EEG data. However, as our main focus is on distinguishing between the groups using task-based data, we have not reported them here but in Supplement Tables III and IV.

### Selecting the most informative subset of depression biomarkers in rs-EEG

**Figure 4:**
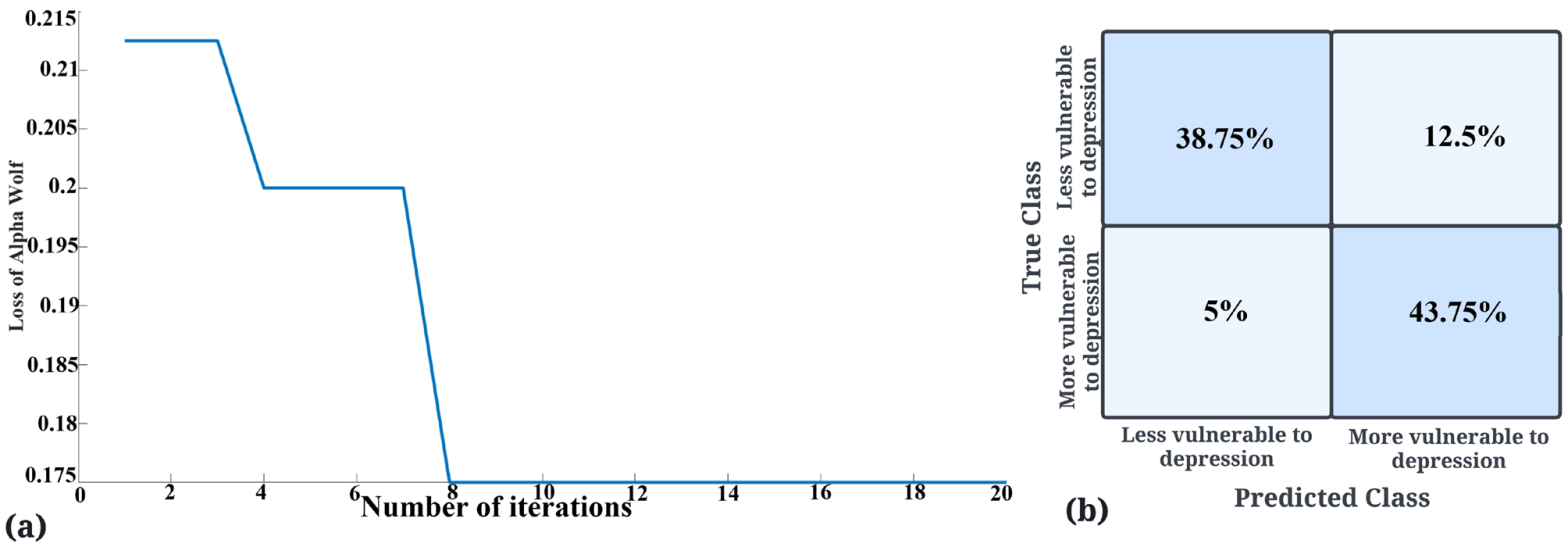
(**a**) Graph showing that the loss of the best wolf decreases over iterations until it converges at iteration 8. (**b**) Confusion matrix for the decision tree classifier that uses GWO for feature selection for the last iteration.

In the previous section, we have seen that depression biomarkers also show significant differences in individuals with high and low vulnerability to depression. However, given a wide range of depression biomarkers, it becomes essential to determine the most informative subset of these biomarkers that can help differentiate between the two groups. We used the biomarkers that had statistically significant channels (Supplement Table I) for feature subset selection. Given that power spectrum EEG data has a huge number of samples than the other biomarkers, we did not incorporate it for this part of the analysis to avoid generating a bias in the classifier. We, however, analysed that data using machine/deep learning and the results are reported in the next section. A decision tree of maximum depth 40 using GWO for feature selection gave 82.75% accuracy with HFD feature in delta and theta frequency and coherence in the gamma frequency band. Figure 4a shows the loss of the model decreasing over iterations and Fig. 4b shows the confusion matrix for the classifier in the last iteration. PSO gave a maximum accuracy of $78.75\%$ with 4 biomarkers, namely, coherence and PLI in gamma wave and correlation in delta and raw EEG data. GA gave the maximum accuracy of $83.5\%$ with minimum of 6 features: coherence in delta and gamma frequency, PLI for raw and gamma frequency and HFD and correlation values in delta frequency. These results suggest that delta and gamma are the main frequencies of interest to differentiate between individuals who are more and less vulnerable to depression. More details on the evolutionary algorithms is in the Supplementary Methods section.

### Machine/deep learning to predict individuals vulnerable to depression using rs-EEG and task-based (SART) EEG signals

Having looked at various aspects of the rs-EEG and its efficacy, we explored how well the task-based measure of “stickiness” could differentiate between the two groups. We also compare which of the two data (rs-EEG and task-based EEG) are more effective prediction.

#### rs-EEG

**Figure 5:**
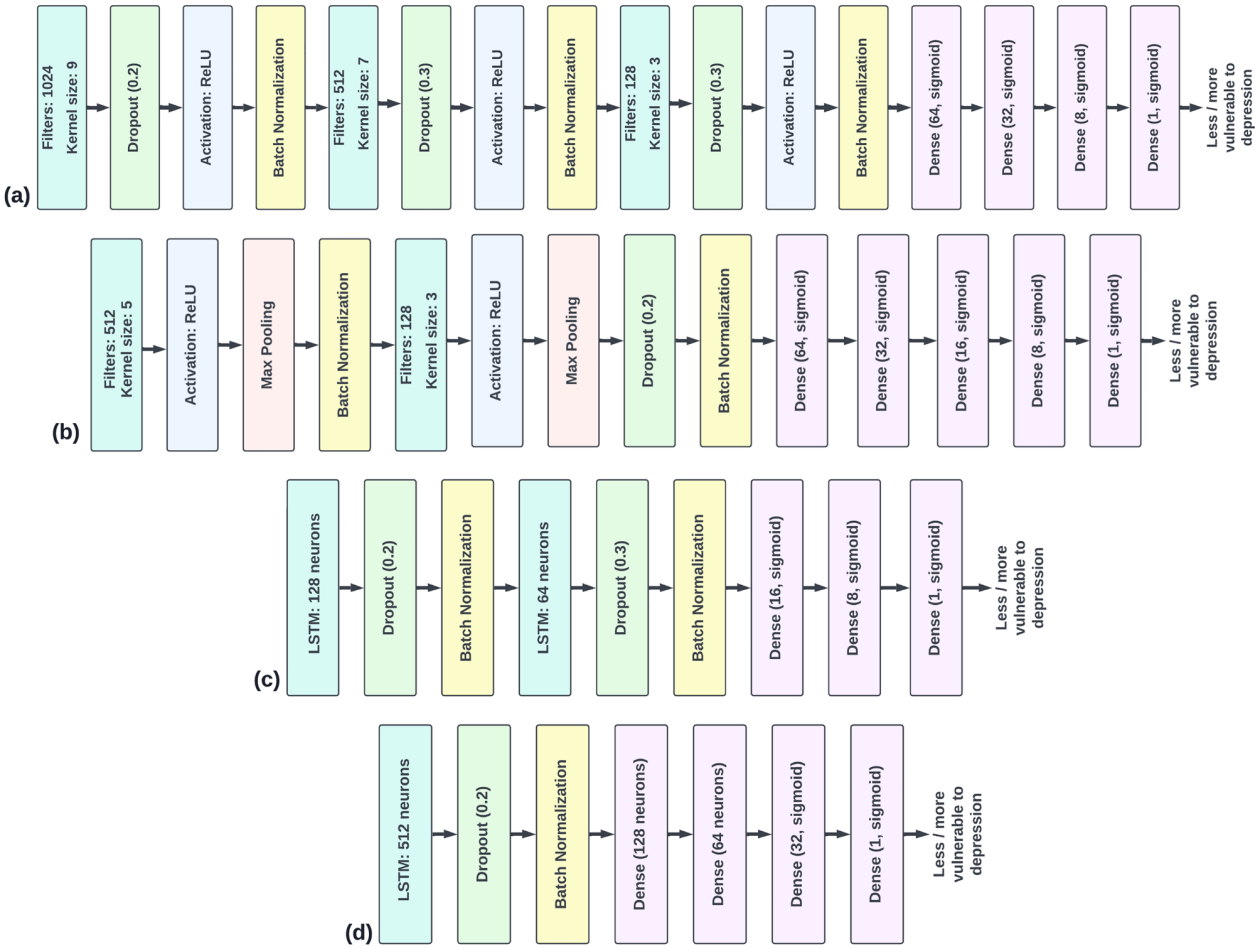
(**a**) 1D-CNN architecture that predicted the high and low vulnerability to depression in individuals with the best accuracy for task-based data. (**b**) 1D-CNN architecture that predicted the high and low vulnerability to depression in individuals with the highest accuracy for rs-EEG data. (**c**) LSTM architecture that predicted the high and low vulnerability to depression in individuals with the best accuracy for rs-EEG data. (**d**) LSTM architecture that predicted the high and low vulnerability to depression in individuals with the best accuracy for task-based data.

**Figure 6:**
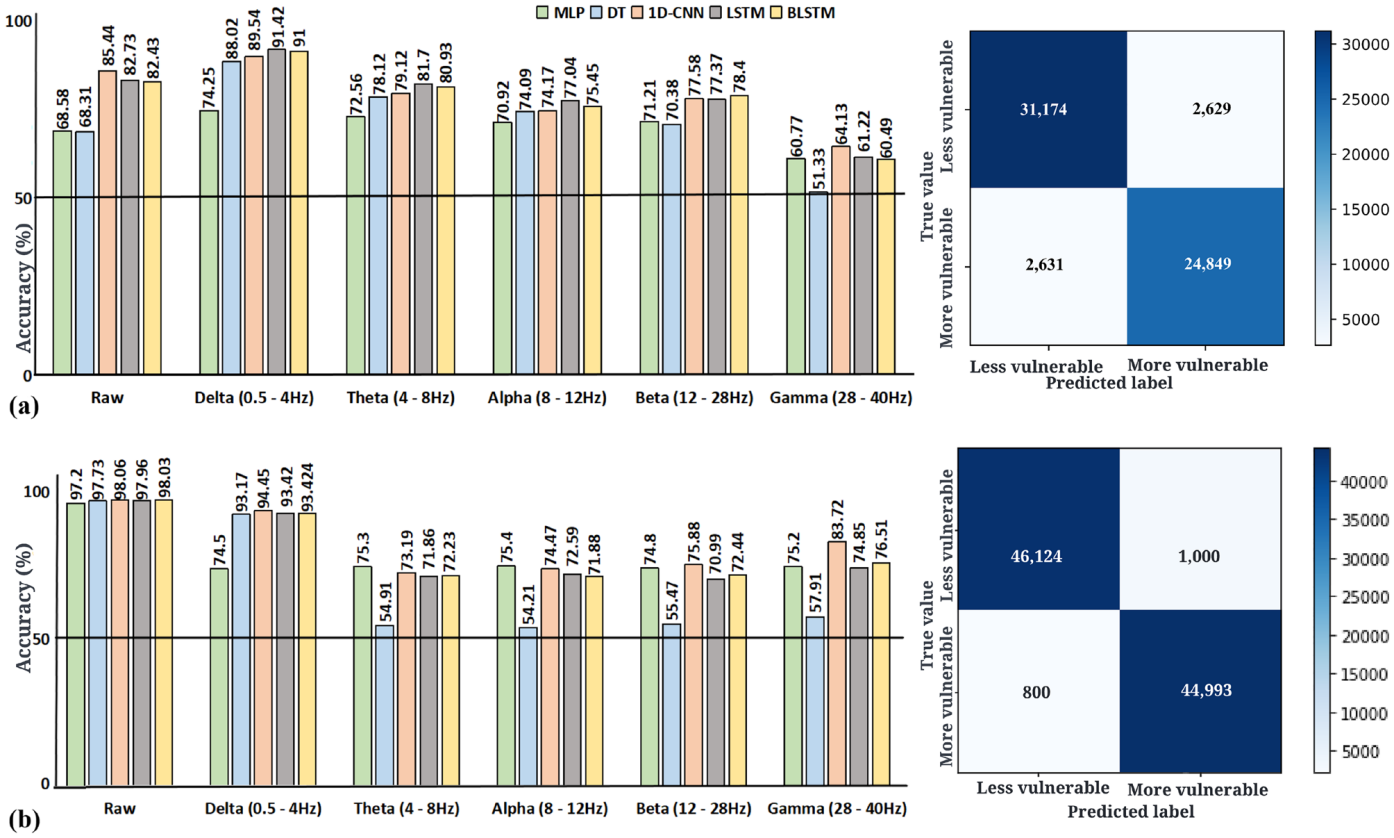
(**a**) Comparison of classification accuracy on the basis of different data aspects for the task-based data test dataset. Delta power was most predictive of vulnerability (high vs low) to depression. The confusion matrix corresponding to test data in the delta band for the dataset using LSTM gave the maximum accuracy out of the various classifiers that were tested. (**b**) Comparison of classification accuracy on the basis of different data aspects for the rs-EEG test dataset. Raw EEG data were most predictive of vulnerability (high vs low) to depression. The confusion matrix corresponding to the test data in raw data for the dataset using 1D-CNN which gave the maximum accuracy out of the various classifiers that were tested.

Here we report the classification results from rs-EEG raw and band power data. A 2-hidden layer neural network with 16 and 8 neurons in each layer, a learning rate of 0.001, batch size 32, and Adam optimiser^47^(beta1 = 0.9 and beta2 = 0.999) gave the highest accuracy of 97.2% for the raw data. DT with a maximum depth of 3 and Gini impurity criterion gave the maximum accuracy of 97.73% in the raw data and 93.17% in the delta brain wave. After experimentation the 1-D CNN (Fig. 5b), and LSTM (Fig. 5c) architecture were found to give the best accuracy of 98.06% and 97.96% for the raw EEG data. BLSTM layer with 256 neurons followed by dropout^48^(0.2) and two fully-connected layers with 64 and 32 neurons, respectively with sigmoid activation gave the maximum accuracy of 98.03% in the raw EEG data. The learning rate, batch size and sequence size for reported models of 1D-CNN, LSTM and BLSTM were 0.001, 32 and 32, respectively. Fig. 6a summarises the results obtained from various classifiers for each brain wave. 1-D CNN and BLSTM predict if an individual is highly or low vulnerable to depression with the highest accuracy of about 98%. Refer to the Supplementary Methods for more information on classifiers.

#### Task-based data

Here we report the classification results from the raw and band power data from the task-based measure of “stickiness”. A 2-hidden layer neural network with 32 and 16 neurons in each layer, a learning rate of 0.001, batch size 32, and Adam optimiser(beta1 = 0.9 and beta2 = 0.999) gave the highest accuracy of 74.25% for the delta wave. DT with a maximum depth of 25 and Gini impurity criterion gave the maximum accuracy of 88.02% in the delta brain wave. After experimentation, the 1-D CNN (Fig. 5a), and LSTM architecture (Fig. 5d) were found to give the best accuracy of 89.54% and 91.42% for the delta brain wave. BLSTM layers with 512 and 256 neurons followed by dropout^48^(0.3), BLSTM layer with 128 neurons and two fully-connected layers with 64 and 16 neurons, respectively gave the maximum accuracy of 91% in the delta wave. The learning rate, batch size and sequence size for reported models of 1D-CNN, LSTM and BLSTM were 0.001, 32 and 32, respectively. Figure 6b summarises the results obtained from various classifiers for each brain wave.

## Discussion

Depression is one of the most prevalent mental disorders that alters the lives of people and levies a huge burden on society. Even though its treatment is available^49^, difficulty in detecting it and the high chances of relapse make it challenging to get rid of it. A lot of research has been done to understand and detect the disorder by finding relevant biomarkers or using machine learning to automatically detect relevant patterns and thus segregate the data into healthy and depressed individuals. Most of the studies have used rs-EEG data or task-based EEG to this effect but not both. In this study, the main question that we focused on is comparing the efficacy of rs-EEG and task-based EEG to differentiate people who are more vulnerable to depression from those who are not. We seek to find a subset of depression markers (rs-EEG) that are more relevant in differentiating between these groups of participants.

Comparing the analysis between rs-EEG and task-based data it was found that the maximum classification accuracy for task-based data (91.42%) is lower than the maximum accuracy obtained from rs-EEG data (98.06%). Hence if one has to look at the primary question of which data will be good for predicting vulnerability to depression, rs-EEG seems to be better than task-based EEG data. However, if one has to understand the mechanisms driving depression, like rumination or stickiness, then task-based data will be more effective.

We found that people who are more vulnerable to depression had, on average, increased activity in the left frontal channel, and decreased activity in the right frontal and occipital channels for raw data (rs-EEG). There is also a high number of electrodes that were found to be statistically significant in differentiating the two groups and amongst the brain waves only delta 0.5–4Hz was found to have significant differences between the two groups. These findings are at odds with most of the studies done with clinically depressed individuals who find theta and low alpha waves to be of more significance^50^. However, there are also a few studies that found delta to be an important contender that showed differences in depressed individuals^51,52^. We found that correlation (raw, delta), coherence (delta, gamma), PLI (raw, delta, and gamma), PLV (raw, delta), and HFD (alpha, delta, and theta) features had electrodes with statistically significant differences between individuals with high and low vulnerability to depression. Various studies have reported HFD in alpha^53,54^, coherence in gamma^55^, PLI for alpha^56^, and PLV for beta bands^57^ as important features. This shows that some biomarkers for depression can be information for vulnerability to depression. On the other hand, biomarkers like DFA, relative gamma power, and alpha asymmetry have been reported as useful by some studies but they did not show any significance in our study. As there exist a lot of depression biomarkers, we used three evolutionary algorithms to determine the most relevant feature subset that can classify if a data sample was from an individual with high or low vulnerability to the depression group. We found HFD, PLI, correlation and coherence features are the most important features. GWO gave the maximum accuracy of 82.75% with HFD and coherence features which is an improvement over the genetic algorithm and PSO. This not only suggests that depression biomarkers can be used for the classification of individuals prone to depression but also indicates that a couple of biomarkers can be used to approach good classification accuracy. If a machine learning model was to be used for this task in real life the model will not have high computation needs.

Since power spectrum values differed from other biomarkers (which had one value per subject), they were used for classification separately. The classification results were corroborated by statistical analysis, which also showed that raw and delta oscillation has the most predictive power. It was found that the 1D-CNN classifier was best at predicting the instances of rs-EEG from individuals more and less vulnerable to depression. It obtained an accuracy of 98% and 94.45% in the raw and delta brain wave respectively. 1D-CNNs have been proven to work very well for predicting time-series data. This reinforces the idea that LSTMs are suitable for similar problems. One thing to note is though the other brain waves perform above chance level, they perform very poorly as compared to delta brain waves.

Entanglement of various factors like age, gender, culture, socio-economic status, education level, etc., in the manifestation and maintenance of depression, makes detection and treatment even more challenging. However, rumination is a core symptom of depression that has been found to generalize across populations. Though rs-EEG is easier to collect than any task-based EEG data, it is possible that task-based data might give more generalizable results. Keeping this perspective in mind we also analyzed “stickiness” data which is a measure of rumination. The statistical test showed that raw, delta and theta brain waves have channels that are statistically different between individuals with high and low vulnerability to depression. Machine learning analysis revealed that BLSTM gave the maximum classification accuracy of 91% for delta brain waves.

However, we should keep in mind that we worked with a limited sample size of forty participants. Further studies with a larger sample size need to be done to corroborate these findings for generalization to a wider population.

## Conclusion

With this study, we tried to draw a comparison between the efficacy of using task-based EEG data vs rs-EEG data for predicting the vulnerability to depression in individuals. It was found that individuals more vulnerable to depression had on average increased activity in the right temporal channel, and decreased activity in the left fronto-central and right occipital channels for raw rs-EEG data. Task-based data showed increased activity in the central part of the brain for individuals with low vulnerability and increased activity in the right temporal, occipital and parietal regions in individuals more vulnerable to depression. It was also found that coherence, PLI, correlation and HFD might be the key biomarkers (rs-EEG) for differentiating the two groups. Though raw rs-EEG data gave higher accuracy (98.06%) than 91.42% for delta waves in task-based data, the performance of classifiers was higher for task-based data across theta, alpha, beta and gamma brain waves. These results open up further avenues for future diagnostics aided by EEG using machine/deep learning.

## Supplementary Information

Supplementary Information 1.

## Data Availability

Data is available at: https://unishare.nl/index.php/s/BDtt49NPkZQDxGy. Scripts are available at: https://github.com/kaushik-pallavi/scripts_vulnerabilityToDepression.
