# Supplementary material for: Comparing resting state and task-based EEG using machine learning to predict vulnerability to depression in a non-clinical population
Source: Sci Rep. 2023 May 8;13:7467. doi: 10.1038/s41598-023-34298-2 (PMC10167316; doi:10.1038/s41598-023-34298-2)
Supplement: Supplementary file 1 — Supplementary Information 1. [file 41598_2023_34298_MOESM1_ESM.pdf]

Supplementary Material: Comparing resting state and task-based EEG using machine learning to predict vulnerability to depression in a non-clinical population

Pallavi Kaushik[1,2,*], Hang Yang[1], Marieke van Vugt[1], and Partha Pratim Roy[2]

[1]Bernoulli Institute of Mathematics, Computer Science and Artificial Intelligence, University of Groningen, 9700 AK, the Netherlands.
[2]Department of Computer Science and Engineering, Indian Institute of Technology, Roorkee, 247667, India
[*]pkaushik@cs.iitr.ac.in/p.kaushik@rug.nl

Legend

Supplemental Table 1: Probe questions asked during the Sustained Attention to Response Task used to determine vulnerability in a task-based context.

Supplemental Table II: Results of statistical analysis on various features of the **resting state EEG data**. Results of statistical analysis on various features of the resting state EEG data. The column 'p-value threshold' contains the p-value threshold corresponding to the False Discovery Rate (FDR) threshold of 0.05. The 'topoplots' column shows topographical maps with the p-values (after FDR correction) in each channel corresponding to the average difference in a given feature for individuals with higher and lower vulnerability to depression.

Supplemental Table III: Results of statistical analysis on the **task-based EEG data**. The column 'p-value threshold' contains the p-value threshold corresponding to the False Discovery Rate (FDR) threshold of 0.05. The 'topoplots' column contains topographical maps with p-values of each channel after FDR. The maps showcase the average difference in EEG activity corresponding to the "stickiness" trials (high and low stickiness combined) between individuals with higher and lower vulnerability to depression.p-value threshold corresponding to the False Discovery Rate (FDR) threshold of 0.05. The 'topoplots' column contains topographical maps with p-values of each channel after FDR. The maps showcase the average difference in EEG activity corresponding to the "stickiness" trials (high and low stickiness combined) between individuals with higher and lower vulnerability to depression.

Supplemental Table IV: Results of statistical analysis on the brain waves of **task-based EEG data**. This table shows the average EEG differences in trials reported as "high/low" stickiness between individuals with higher and lower vulnerability to depression. The column 'p-value threshold' contains the p-value threshold corresponding to the False Discovery Rate (FDR) threshold of 0.05.

Supplemental Table V: Results of statistical analysis on the brain waves of **task-based EEG data**. This table shows the average differences in EEG activity between "high" and "low" stickiness trials for individuals within the two groups. The column 'p-value threshold' contains the p-value threshold corresponding to the False Discovery Rate (FDR) threshold of 0.05.

Supplemental Methods: Background on Classifiers

Supplemental Methods: Hyperparameter tuning

Supplemental Methods: Evolutionary algorithms

Supplemental Methods: Feature Extraction

Supplemental Table VI: The confusion matrices for **rs-EEG classification** results using 1D-CNN, LSTM and BLSTM

Supplemental Table VII: The confusion matrices for classification results of **task-based EEG data** using 1D-CNN, LSTM and BLSTM

Supplemental Table VIII: The confusion matrices for classification results of **task-based EEG data** using MultiLayer Perceptron (MLP) and Decision Tree (DT)

Supplemental Table IX: The confusion matrices for classification results of **rs-EEG** using MultiLayer Perceptron (MLP) and Decision Tree (DT).

Supplemental Table 1. Probe questions asked during the Sustained Attention to Response Task used to determine vulnerability in a task-based context.

Dimension of the thoughts	Probe questions	Responses
Content	What were you thinking?	1. I was completely focused on the task.<br>2. I was evaluating aspects of the task (e.g., how I was doing or how long the task was taking).<br>3. I was thinking about personal things.<br>4. I was distracted by my environment (e.g., sound, temperature, my physical state).<br>5. I was daydreaming or thinking about task-irrelevant things.<br>6. I was not paying attention, and did not think about anything in particular.
Degree of self-focus	To what extent were your thoughts self-focused?	Ranging from 1 (completely non self-focused/about others) to 9)completely self-focused)
Valence	How positive or negative were your thoughts?	Ranging from 1 (very negative) to 9 (very positive)
Stickiness	How difficult was it to disengage from the thoughts?	Ranging from 1 (very easy) to 9 (very difficult)

Supplemental Table II. Results of statistical analysis on various features of the resting state EEG data. The column ‘p-value threshold’ contains the p-value threshold corresponding to the False Discovery Rate (FDR) threshold of 0.05. The ‘topoplots’ column shows topographical maps with the p-values (after FDR correction) in each channel corresponding to the average difference in a given feature for individuals with higher and lower vulnerability to depression.

	Feature	p-value threshold corresponding to FDR value of 0.05	Topoplot

1	Raw EEG data after ICA	4.880e-17	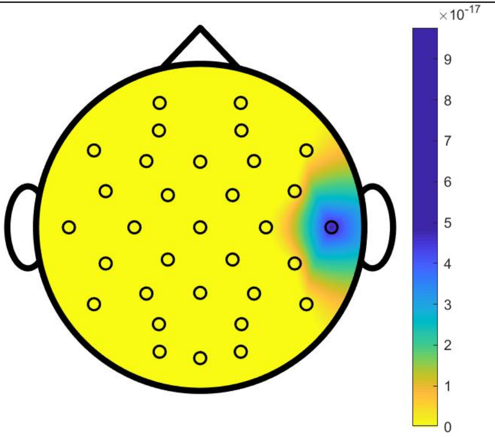
	Power spectrum		
2	Delta brain wave (0.5-4Hz)	1.095e-11	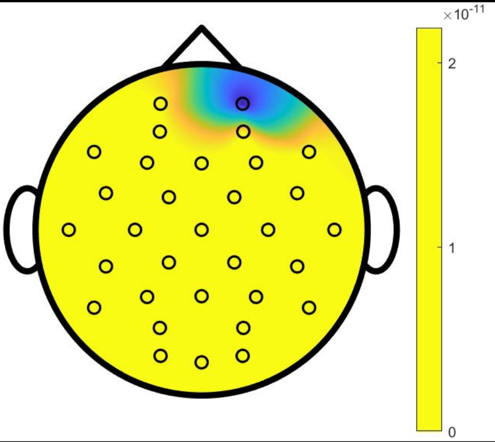
3	Theta brain wave (4-8Hz)	0	No channel significant
4	Alpha brain wave (8-12Hz)	0	No channel significant
5	Beta brain wave 12-28Hz)	0	No channel significant
6	Gamma brain wave(28-40Hz)	0	No channel significant
	Correlation		

7	Raw EEG data after ICA	2.570e-04	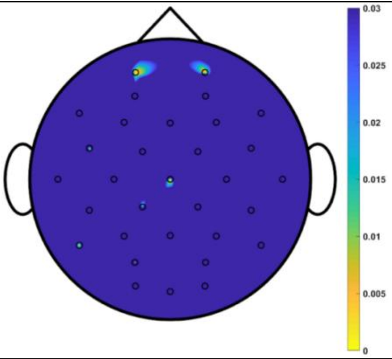
8	Delta brain wave (0.5-4Hz)	0.014	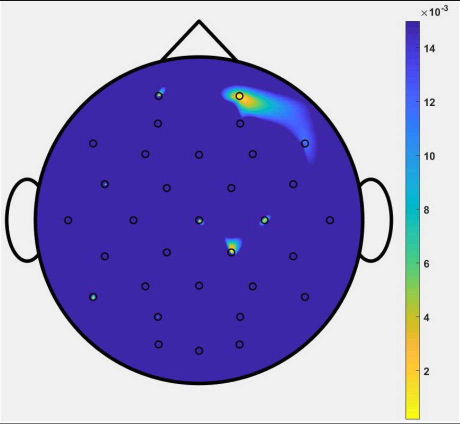
9	Theta brain wave (4-8Hz)	0	Nothing significant
10	Alpha brain wave (8-12Hz)	0	Nothing significant
11	Beta brain wave 12-28Hz)	0	Nothing significant
12	Gamma brain wave(28-40Hz)	0	Nothing significant
	Absolute value of Coherence		
13	Raw EEG data after ICA		Nothing significant
14	Delta brain wave (0.5-4Hz)	0.008	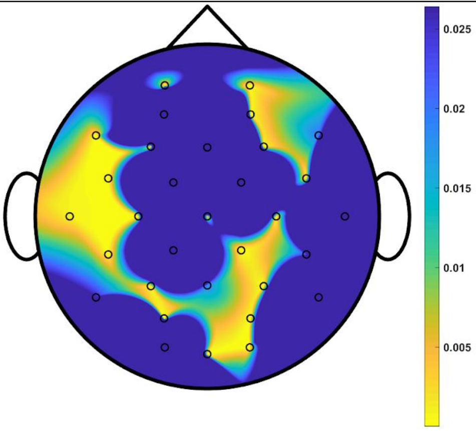
15	Theta brain wave (4-8Hz)	0	Nothing significant
16	Alpha brain wave (8-12Hz)	0	Nothing significant
17	Beta brain wave 12-28Hz)	0	Nothing significant

18	Gamma brain wave(28-40Hz)	0.002	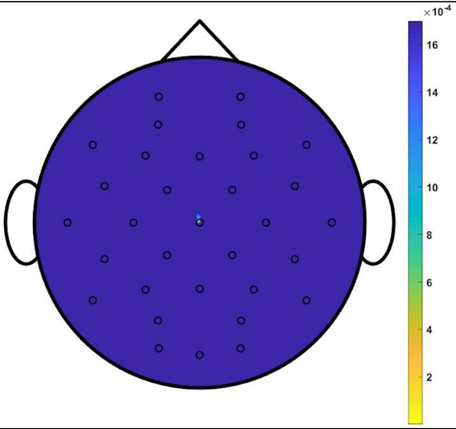
	Imaginary part of coherence		
19	Raw EEG data after ICA	0	Nothing significant
20	Delta brain wave (0.5-4Hz)	0	Nothing significant
21	Theta brain wave (4-8Hz)	0	Nothing significant
22	Alpha brain wave (8-12Hz)	0	Nothing significant
23	Beta brain wave 12-28Hz)	0	Nothing significant
24	Gamma brain wave(28-40Hz)	0	Nothing significant
	Phase lag index		
25	Raw EEG data after ICA	0.004	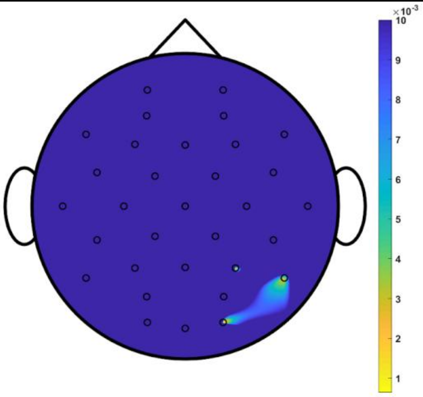
26	Delta brain wave (0.5-4Hz)	0.014	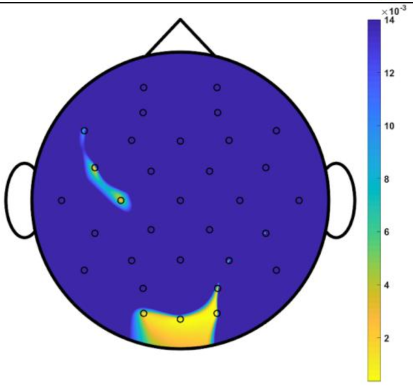
27	Theta brain wave (4-8Hz)	0	Nothing significant
28	Alpha brain	0	Nothing significant

	wave (8-12Hz)		
29	Beta brain wave 12-28Hz)	0	Nothing significant
30	Gamma brain wave(28-40Hz)	0.004	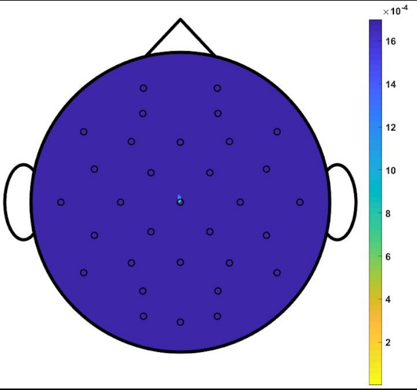
	Phase lag value		
31	Raw EEG data after ICA	0.026	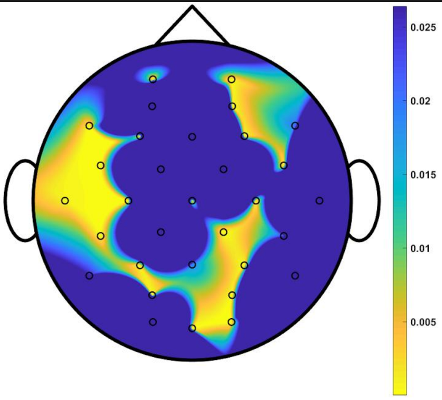
32	Delta brain wave (0.5-4Hz)	0.029	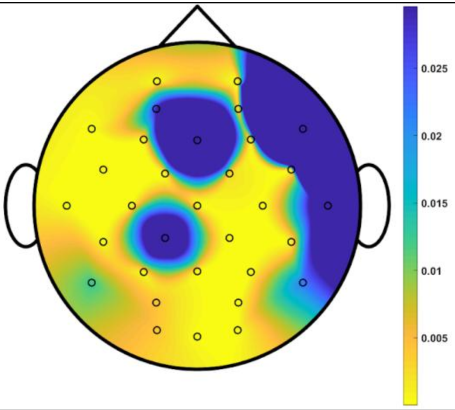
33	Theta brain wave (4-8Hz)	0	Nothing significant
34	Alpha brain wave (8-12Hz)	0	Nothing significant
35	Beta brain wave 12-28Hz)	0	Nothing significant
36	Gamma brain wave(28-40Hz)	0	Nothing significant
37	Alpha asymmetry		
		0	Nothing significant
38	Relative gamma		
		0	Nothing significant

	Huguchi fractal dimension		
39	Raw EEG data after ICA	0	Nothing significant
40	Delta brain wave (0.5-4Hz)	0.008	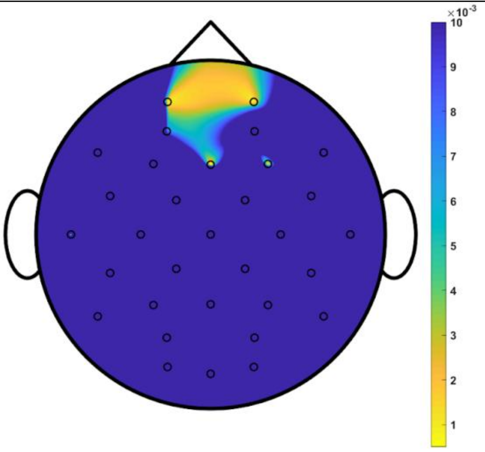
41	Theta brain wave (4-8Hz)	0.011	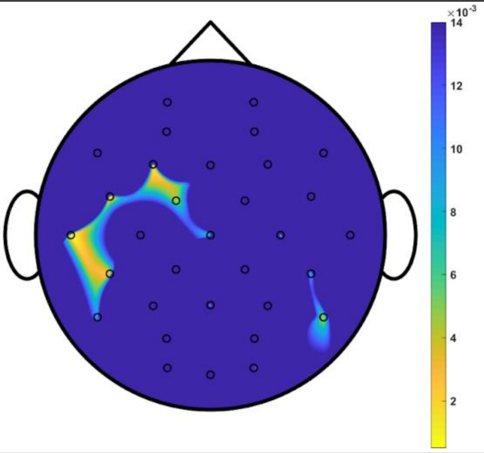
42	Alpha brain wave (8-12Hz)	0.007	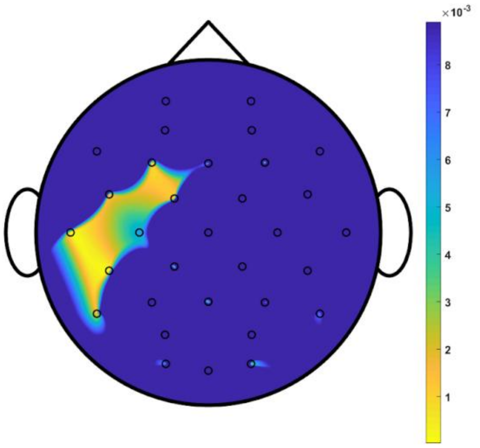
43	Beta brain wave 12-28Hz)	0	Nothing significant
44	Gamma brain wave(28-40Hz)	0	Nothing significant
	Detrended fluctuation analysis		
45	Raw EEG data after ICA	0	Nothing significant
46	Delta brain	0	Nothing significant

	wave (0.5-4Hz)		
47	Theta brain wave (4-8Hz)	0	Nothing significant
48	Alpha brain wave (8-12Hz)	0	Nothing significant
49	Beta brain wave 12-28Hz)	0	Nothing significant
50	Gamma brain wave(28-40Hz)	0	Nothing significant
	Theta Asymmetry		
51		0	Nothing significant

Supplemental Table III. Results of statistical analysis on the **task-based EEG data**. The column ‘p-value threshold’ contains the p-value threshold corresponding to the False Discovery Rate (FDR) threshold of 0.05. The ‘topoplots’ column contains topographical maps with p-values of each channel after FDR. The maps showcase the average difference in EEG activity corresponding to the “stickiness” trials (high and low stickiness combined) between individuals with higher and lower vulnerability to depression.p-value threshold corresponding to the False Discovery Rate (FDR) threshold of 0.05. The ‘topoplots’ column contains topographical maps with p-values of each channel after FDR. The maps showcase the average difference in EEG activity corresponding to the “stickiness” trials (high and low stickiness combined) between individuals with higher and lower vulnerability to depression.

**Feature**	**p-value threshold for FDR value of 0.05**	**Topoplots**
Raw EEG (data after ICA)	2.155e-04	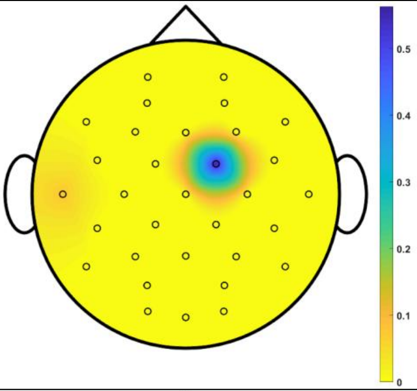
Delta brain wave (0.5-4Hz)	0.017	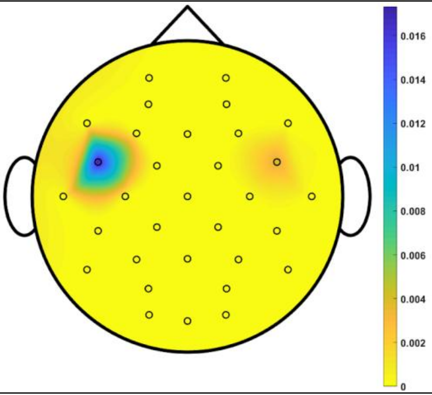

Theta brain wave (4-8Hz)	0.029	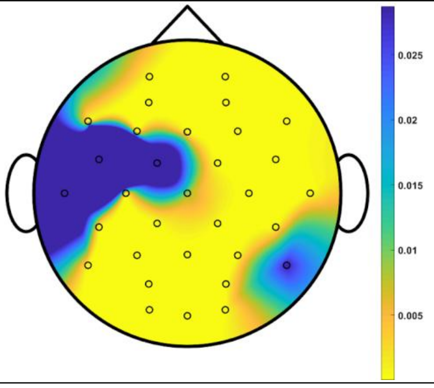
Alpha brain wave (8-12Hz)	0	Nothing significant
Beta brain wave 12-28Hz)	0	Nothing significant
Gamma brain wave(28-40Hz)	0	Nothing significant

Supplement Table IV. Results of statistical analysis on the brain waves of **task-based EEG data**. This table shows the average EEG differences in trials reported as "high/low" stickiness between individuals with higher and lower vulnerability to depression. The column 'p-value threshold' contains the p-value threshold corresponding to the False Discovery Rate (FDR) threshold of 0.05.

**Feature**<br>Mean difference in the two groups based on the EEG data from the reported high stickiness trials	**p-value threshold for FDR of 0.05**	**Topoplot**
Raw EEG data after ICA	0.018	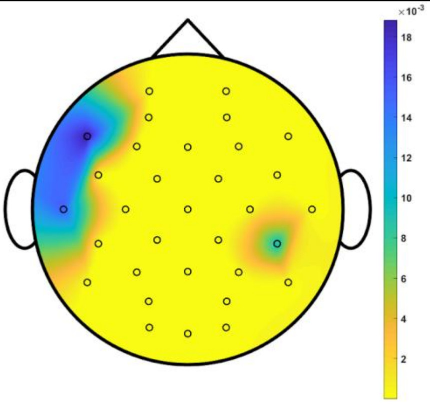
Delta brain wave (0.5-4Hz)	0.003	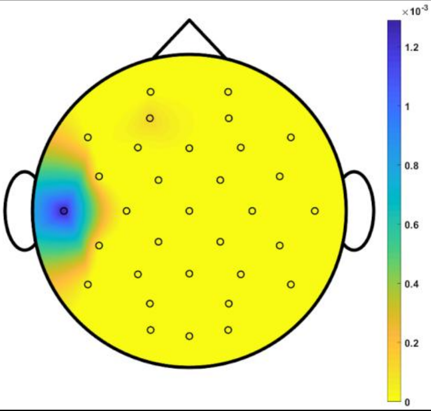

Theta brain wave (4-8Hz)	0.014	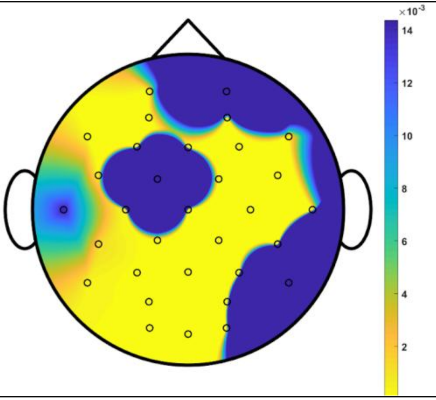
Alpha brain wave (8-12Hz)	-	Nothing significant
Beta brain wave 12-28Hz)	-	Nothing significant
Gamma brain wave(28-40Hz)	-	Nothing significant
**Feature**<br>Mean difference in the two groups based on the EEG data from reported low stickiness trials	**p-value threshold for FDR of 0.05**	**Topoplot**
Raw EEG data after ICA	0.012	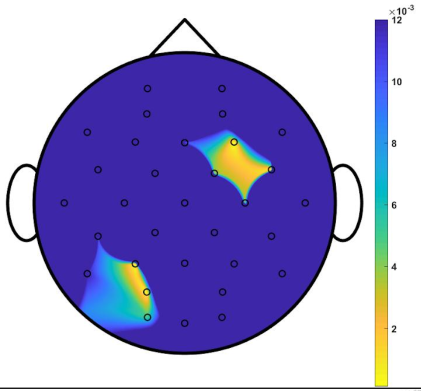
Delta brain wave (0.5-4Hz)	6.421e-37	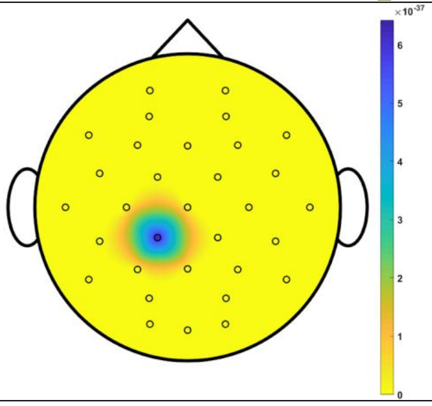

Theta brain wave (4-8Hz)	0.038	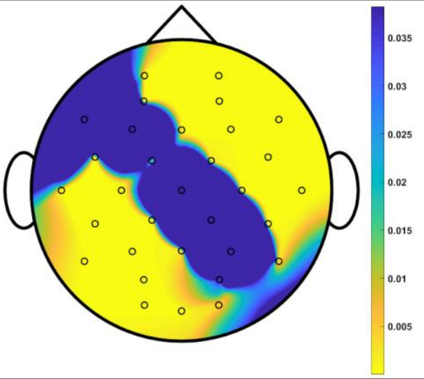
Alpha brain wave (8-12Hz)	0.012	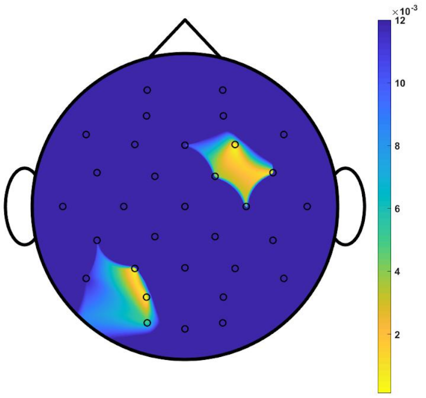
Beta brain wave 12-28Hz)	-	Nothing significant
Gamma brain wave(28-40Hz)	-	Nothing significant

Supplement Table V. Results of statistical analysis on the brain waves of **task-based EEG data**. This table shows the average differences in EEG activity between "high" and "low" stickiness trials for individuals within the two groups. The column 'p-value threshold' contains the p-value threshold corresponding to the False Discovery Rate (FDR) threshold of 0.05.

**S.no.**	**Feature**<br>Mean difference in EEG activity corresponding to high vs low stickiness for individuals more vulnerable to depression	**p-value threshold for FDR of 0.05**	**Topoplot**
1	Raw EEG data after ICA	0.043	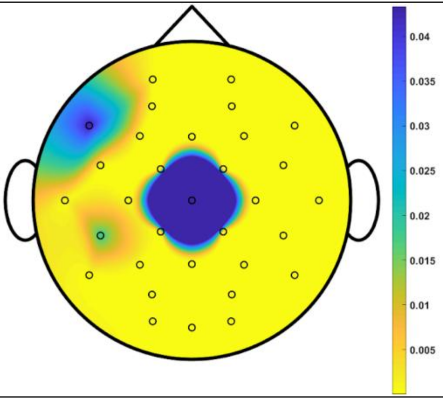

2	Delta brain wave (0.5-4Hz)	0.007	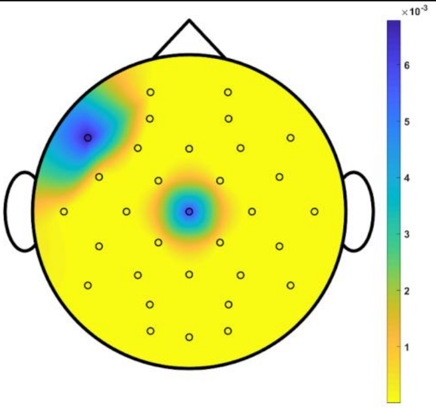
3	Theta brain wave (4-8Hz)	0.015	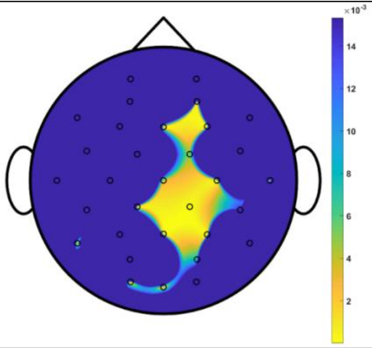
4	Alpha brain wave (8-12Hz)	3.632e-04	Nothing significant
5	Beta brain wave 12-28Hz)	-	Nothing significant
6	Gamma brain wave(28-40Hz)	-	Nothing significant
**S.no.**	**Feature** Mean difference in EEG activity corresponding to high vs low stickiness for individuals less vulnerable to depression	**p-value threshold for FDR of 0.05**	**Topoplot**
1	Raw EEG data after ICA	1.281e-08	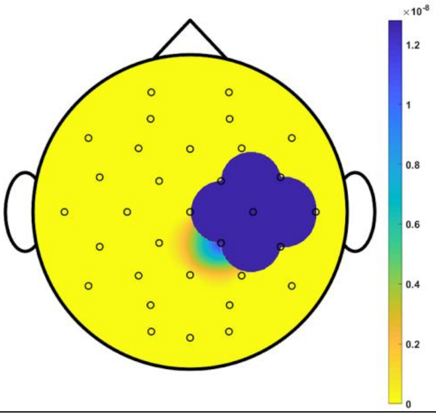

2	Delta brain wave (0.5-4Hz)	0.005	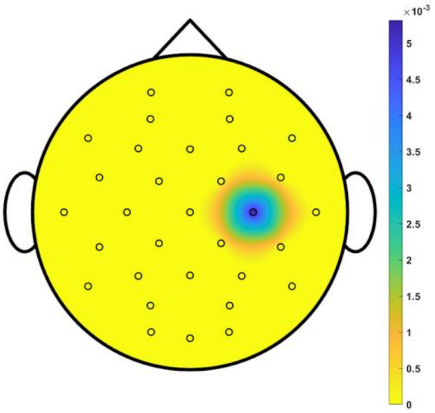
3	Theta brain wave (4-8Hz)	0.003	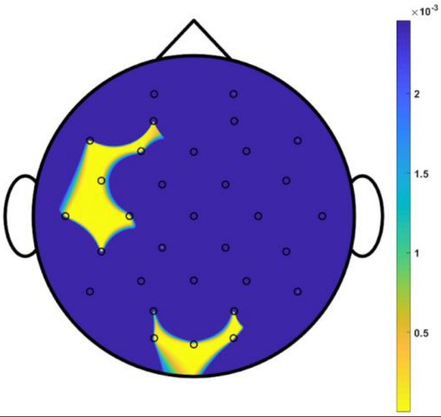
4	Alpha brain wave (8-12Hz)	0.016	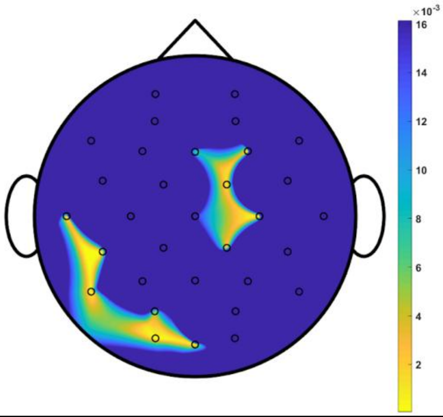
5	Beta brain wave 12-28Hz)	-	Nothing significant
6	Gamma brain wave(28-40Hz)	-	Nothing significant

Supplement Methods: Background on Classifiers

Support Vector Machine (SVM)

It is a popular supervised learning algorithm that is used both for classification and regression. SVM plots the data points (x_1, x_2, ..., x_n, 'n' being the number of features) in an n-dimensional space and finds a hyperplane or a set of hyperplanes that separates the support vectors by maximum distance. For handling non-linear data, kernel functions are beneficial. They allow mapping non-linear data to a higher dimension where they are linearly separable, which can then be classified by SVM.

MultiLayer Perception (MLP)

This classifier comprises of an input layer consisting of 'n+1' neurons (data (x_1,x_2, ..., x_n) and a bias 'b'), 'i' hidden layer neurons and 'c' output layer neurons ('c' being the total classes in the dataset). Each neuron in a layer is a weighted sum of inputs from the previous layer followed by a non-linear activation function, eg for Suppl Fig. 1 ($h_i = \tanh(w_1*x_1+w_2*x_2+...+w_n*x_n+b_1*w_0)$).

Output (Y) is determined at the last layer and error or loss is evaluated using the ground truth (Y') for each data point. Backpropagation algorithm [1] uses these errors to train MLP i.e. finetune the weights to optimize classification performance.

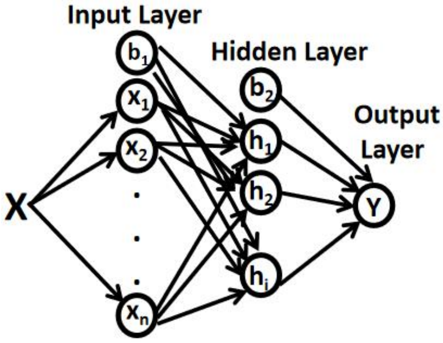

Suppl Figure 1. A multilayer perceptron

Random Forest (RF)

It is an ensemble technique that makes use of many decision trees for classification. Many decision trees are trained independently and the final decision of the RF is based on consensus. During the training phase, each decision tree's training set is created by random sampling. Addition of more decision trees decreases the variance in the model, however if the correlation between any decision trees increases, the error rate of the model also increases [2]. Thus, number of decision trees forming a forest is also a hyperparameter. Using many decision trees for classification, a RF classifier is good at dealing with noisy and missing data.

1D - Convolutional Neural Network (CNN)

CNNs are rooted in image processing as they were primarily used for image classification. They are known to capture the spatial features in data using a kernel that makes it robust at detecting distribution of colours, detect edges, etc. However they are no longer limited to handling images. 1d - CNNs, are suitable to work with time series data as their kernel moves along one dimension. CNNs typically consist of a convolutional layer (learns the features from the input), pooling layer (reduces the size of the feature maps while preserving important features), and fully connected layers (connecting the previous layer to the output neurons).

Long Short Term Memory (LSTM)

It is a recurrent neural network that tracks long-term dependencies in the input data leading it to predict the time-series data well. A basic LSTM unit consists of memory cells where each cell comprises of 3 gates, namely input, output and forget gate. The cell is responsible for handling the long term dependency while the three gates regulates the flow of values between the different layers of the LSTM network. The basic architecture of a single LSTM cell is as shown in (Suppl Fig. 2) , where x^t, c^t, h^t, c^{t-1} and h^{t-1} represent the previous input, current cell state, current output, previous cell state and the previous output, respectively. σ represents the sigmoid activation function, whereas '*' and '+' represent the mathematical operations of multiplication and addition, respectively. f^t, i^t and o^t represent the forget, input and output gate respectively.

The forget gate decides what previous information should be forgotten. Its output is formalised in Suppl equation (1).

$$f_t=\sigma(x_t*U_f+h_{t-1}*w_f) \qquad \text{Suppl (1)}$$

Where U_f and w_f denote the weight associated with the input and hidden state respectively.

The input gate is used to quantify the importance of the new information brought in by the input. It is represented by Suppl equation (2).

$$i_t=\sigma(x_t*U_i+h_{t-1}*w_i) \qquad \text{Suppl (2)}$$

U_i and w_i represent the weights associated with the input and the hidden state respectively.

The output gate is represented by Suppl equation (3).

$$o_t=\sigma(x_t*U_o+h_{t-1}*w_o) \qquad \text{Suppl (3)}$$

U_o and w_o represent the weights associated with the input and the hidden state respectively

The current hidden state h_t is given by Suppl equation (4)

$$h_t=\tanh(c_t)*o_t \qquad \text{Suppl (4)}$$

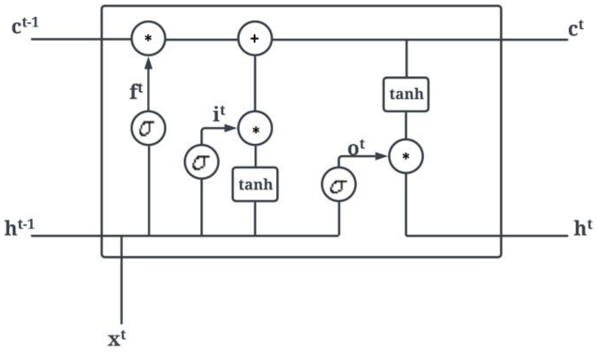

Suppl Figure 2. A LSTM cell in our network

Supplement Methods: Hyperparameter tuning

1. We tried running various models with different permutations of layers, regularizers, optimizers, batch size, window size and activation function. Details of the experiments conducted for machine/deep learning:

LSTM:
1. Layers: LSTM layers ranging from 1-5 with units in each layer ranging from 1024-32. Each consequent layer had a step down in power of 2 for the units. For example if the first layer had 512 units, the consequent LSTM layers will have 256, 64, 32, and 16 units respectively for 5 layered LSTM. For a four layered LSTM model we experimented with 256, 64, 32 and 16 units in each layer respectively. Similarly we experimented with addition of 3-1 dense layers with neurons ranging from 16, 8, and 4.
2. Timestep/window size of 64, 32 and 16.
3. Dropout rates of 0.5, 0.4, 0.3, and 0.2
4. Inclusion and exclusion of batch normalization
5. RMSProp and Adam optimizers
6. Activations 'tanh' and 'sigmoid' for the dense layers.

CNN:
7. CNN layers ranging from 3-1 with 1024-64 filters and kernel size of 5 and 7.
8. Timestep/window size of 64, 32 and 16.
9. Dropout rates of 0.5, 0.4, 0.3, and 0.2
10. Inclusion and exclusion of batch normalization
11. RMSProp and Adam optimizers
12. Activations 'tanh' and 'sigmoid' for the dense layers.

DT:
1. Number of decision trees ranging from 60-10 and their maximum depth ranging from 32-3

MLP:
1. A 2, 3-layer MLP with neurons in each layer ranging from 64-4.
2. Batch size of 64 and 32.
3. SGD and Adam optimisers.
4. Learning rate: 0.001, 0.0001

Supplement Methods: Evolutionary Algorithms
Particle Swarm Optimization (PSO)
- Number of particles: 10
-Maximum iterations:40
-Transfer function, T(x):

$$T(x) = \left| \frac{2}{\pi} \arctan\left(\frac{\pi}{2} x \right) \right|$$

This transfer function gave the best results in [4] and hence has been used for our work as well.

Gray Wolf Optimization(GWO)

- Number of wolves: 50
- Maximum iterations: 100
-Transfer function, T(x):

$$\frac{1}{1+e^{-10*X-0.5}}$$

This transfer function, mentioned as $bGWO_2$ in [3] gave the best results for binary classification using GWO and hence, has been used for this study as well.

Genetic Algorithm (GA)

-Number of genes: 150
-Maximum iterations: 30
-Crossover rate: 0.8
-Mutation rate: 0.1
-Elite rate: 0.2
-Roulette selection

Supplement Methods: Feature Extraction

Discrete wavelet transform:

The DWT is done by obtaining the Approximation (A) and Detail (D) coefficients by performing decomposition at various levels. It is done through the use of low and high pass filters at various levels. The low pass filter (L) ignores the high-frequency fluctuations and helps in preserving the low-frequency trends. Similarly, the high pass filter (H) helps in keeping the high-frequency fluctuations and ignoring the slow trends in the signal. The outcomes from the low pass filters help in forming the approximation coefficients, and those from the high pass filters help in forming the detailed coefficients. The Daubechies (Db)-8 wavelet transform has been used in this work. Hence the levels of decomposition are 8.

$$\int_{-\infty}^{\infty} \psi(t)\, dt = 0 \tag{1}$$

$$\psi_{m,n}(t) = a_0^{-m/2}\psi(a_0^m t - nb_0) \tag{2}$$

The scaling and wavelet functions required for the evaluation A and D coefficients are given in (3) and (4), respectively.

$$\phi_{p,q}(t) = 2^{p/2}h(2^p t - q) \tag{3}$$

$$\omega_{p,q}(t) = 2^{p/2}g(2^p t - q) \tag{4}$$

The A_i and D_i coefficients at the ith level are evaluated using (5) and (6), respectively.

$$A_i = \frac{1}{\sqrt{T}} \sum_t x(t)\phi_{p,q}(t) \tag{5}$$

$$D_i = \frac{1}{\sqrt{T}} \sum_t x(t)\omega_{p,q}(t) \tag{6}$$

The Db-8 decomposition gives the remaining four wavelet coefficients that correspond to noise and five wavelet coefficients corresponding to alpha, beta, delta, gamma and theta brain waves.

Correlation

The Pearson correlation coefficient assesses the extent of linear dependency between two electrode channels in the time domain. It is one of the simplest metrics for non-directed model-based interactions. It is mathematically defined as:

$$Corr_{xy} = \frac{Cov(x,y)}{\sigma_x \sigma_y} \tag{7}$$

where Cov(x, y) denotes the co-variance between electrodes x and y, and σx and σy denote the electrodes' standard deviations, respectively. The higher the absolute value of Corrxy, the stronger will be the correlation.

Coherence

It is a widely used metric used to quantify phase synchronicity between a pair of signals. It is the spectral cross-correlation between them, normalized by their power spectrum. The value of coherence defines the level of linear interdependence between two signals. It is mathematically computed as:

$$Coh_{xy}(f) = \frac{|\frac{1}{n}\sum_{k=1}^{n} Ax(f,k)Ay(f,k)e^{i(\varphi_x(f,k)-\varphi_y(f,k))}|}{\sqrt{\left(\frac{1}{n}\sum_{k=1}^{n} A_x^2(f,k)\right)\left(\frac{1}{n}\sum_{k=1}^{n} A_y^2(f,k)\right)}} \quad (8)$$

where n symbolizes the number of data points in an experiment, A is the amplitude, and φ is the phase of the signal. The numerator term on a single trial is represented by the cross-spectral density of the signals x and y at frequency f. The square root of the product of the power estimations on a single trial of the signals x and y at frequency f is represented in the denominator. It can be concisely represented as:

$$Coh_{xy}(f) = \frac{|Sxy(f)|}{\sqrt{Sxx(f)Syy(f)}} \quad (9)$$

where Sxy(f) is the signal's cross-spectral density, and Sxx(f) and Syy(f) are the signal's power spectral density, respectively.

Phase Locking Value

PLV can be defined as the coherence of amplitude-normalized Fourier-transformed signals. It is based on the assumption that signal amplitude and phase are statistically independent. Hence, only phase synchronization is employed to determine a probable functional connection between two channels of EEG recordings. The value of PLV can be obtained by putting Ax(.,.) = Ay(.,.) = 1 in the Eq. (8):

$$PLV_{xy}(f) = \frac{|\frac{1}{n}\sum_{k=1}^{n} 1x(f,k)1y(f,k)e^{i(\varphi_x(f,k)-\varphi_y(f,k))}|}{\sqrt{\left(\frac{1}{n}\sum_{k=1}^{n} 1_x^2(f,k)\right)\left(\frac{1}{n}\sum_{k=1}^{n} 1_y^2(f,k)\right)}} \quad (10)$$

$$= |\frac{1}{n}\sum_{k=1}^{n} e^{i(\varphi_x(f,k)-\varphi_y(f,k))}| \quad (11)$$

PLV has a value between 0 and 1, with 0 indicating no synchronization and 1 indicating full-phase synchronization.

Phase Lag Index

PLI is a metric for the asymmetry of phase difference distributions between two EEG signals. By ignoring zero and π phase disparities, it aims at minimizing the effect of volume conduction in phase synchronization measurement. It determines the asymmetry of the distribution of instantaneous phase differences using the Hilbert transformation. A time series of phase differences Δφ (tk), k = 1... N can be used to calculate an index of the asymmetry of the phase difference distribution using:

$$PLI_{xy}(f) = |\frac{1}{n}\sum_{k=1}^{n} sign[\Delta\phi t_k]| \quad (12)$$

The PLIxy(f) value ranges from 0 to 1, with 1 denoting perfect phase synchronization and 0 denoting no coupling or coupling with a phase discrepancy centered around 0 mod π.

Supplement Results

Supplement Table VI. The confusion matrices for **rs-EEG classification** results using 1D-CNN, LSTM and BLSTM results using 1D-CNN, LSTM and BLSTM

<table>
<tr><th></th><th>1D-CNN</th><th>LSTM</th><th>BLSTM</th></tr>
<tr><td>Raw</td>
<td>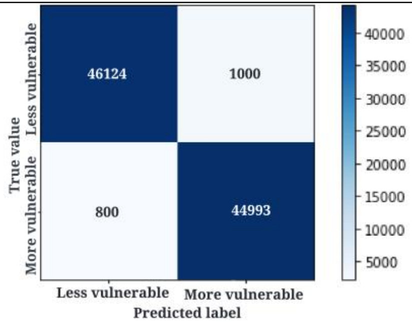
</td>
<td>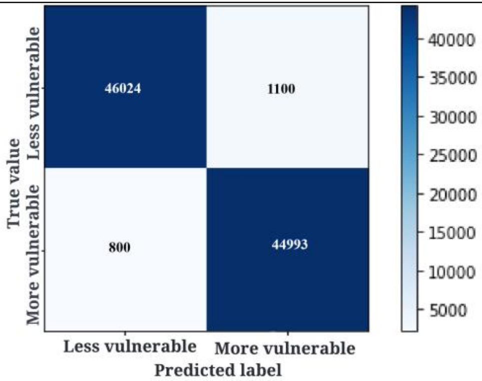
</td>
<td>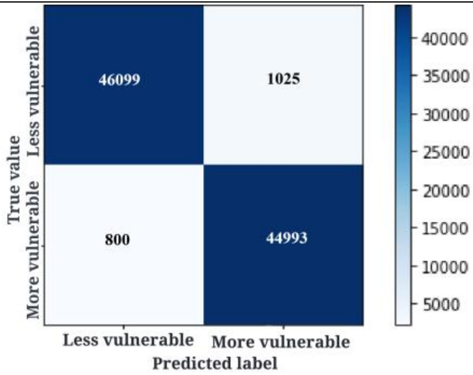
</td></tr>
<tr><td>Delta (0.5-4 Hz)</td>
<td>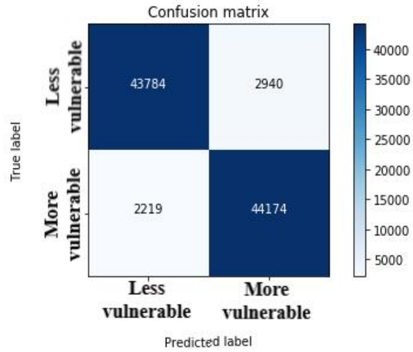
</td>
<td>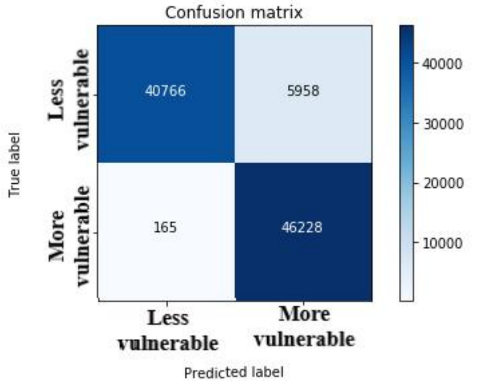
</td>
<td>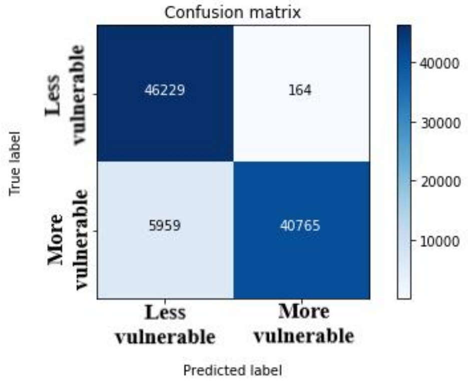
</td></tr>
<tr><td>Theta (4-8 Hz)</td>
<td>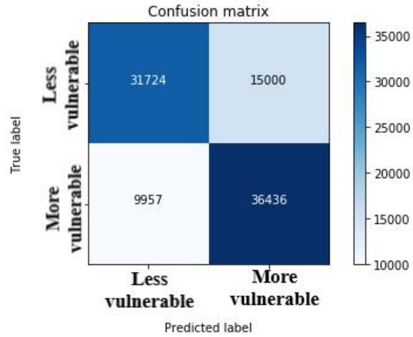
</td>
<td>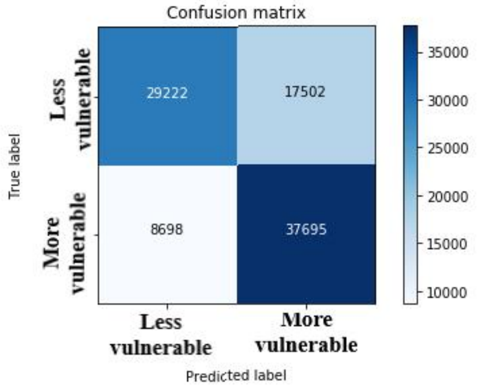
</td>
<td>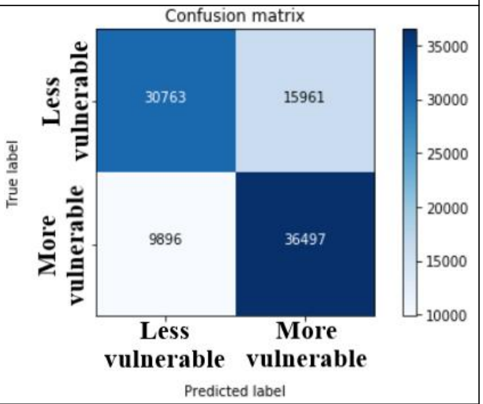
</td></tr>
<tr><td>Alpha (8-12 Hz)</td>
<td>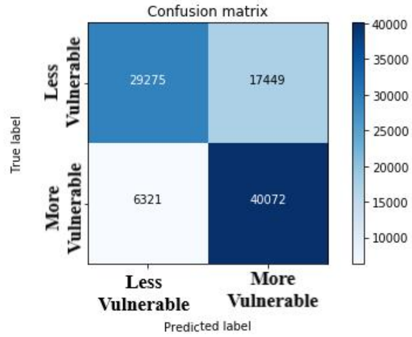
</td>
<td>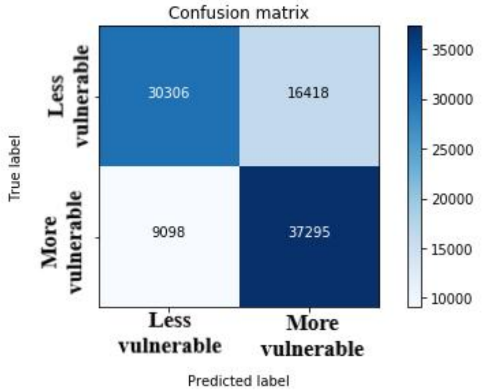
</td>
<td>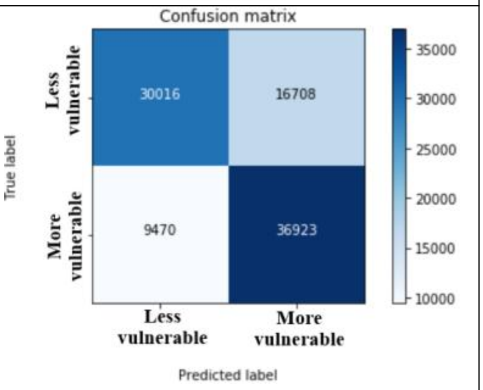
</td></tr>
</table>

**Beta (12-28 Hz)**	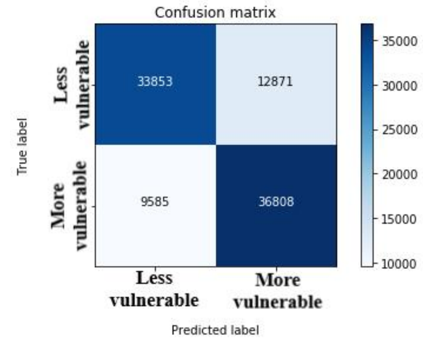	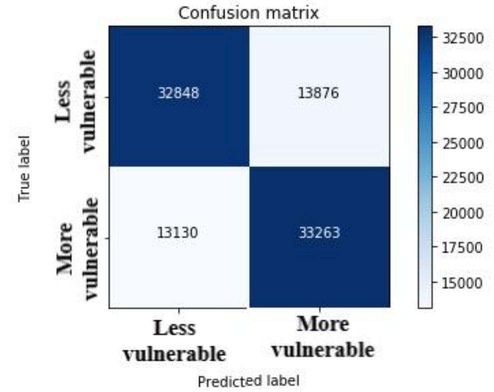	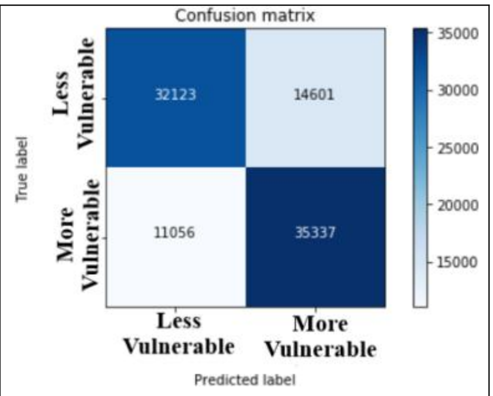
**Gamma (28-40 Hz)**	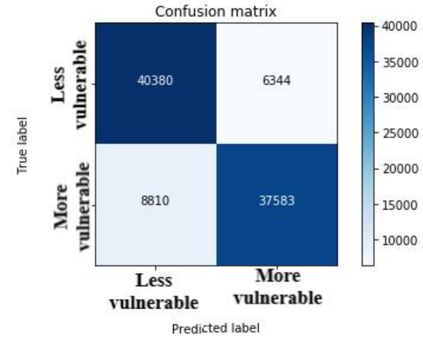	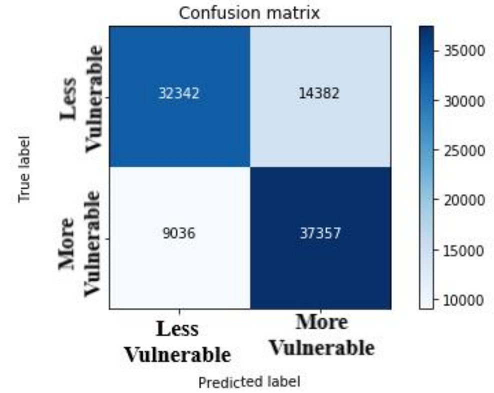	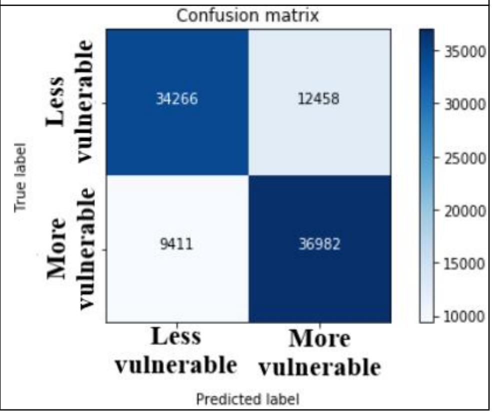

Supplement Table VII. The confusion matrices for classification results of **task-based EEG data** using 1D-CNN, LSTM and BLSTM

	**1D-CNN**	**LSTM**	**BLSTM**
**Raw**	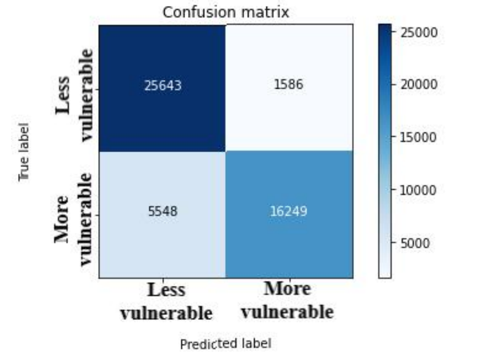	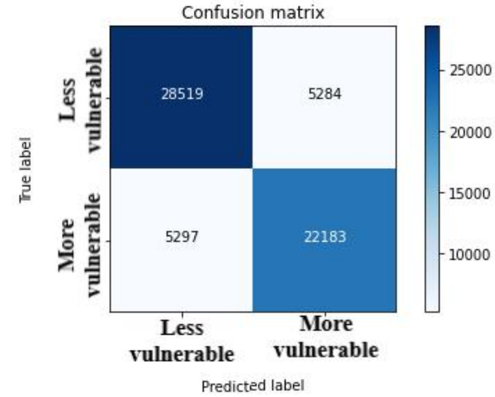	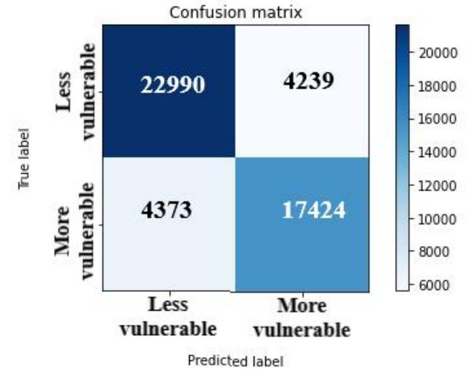

**Delta (0.5-4 Hz)**	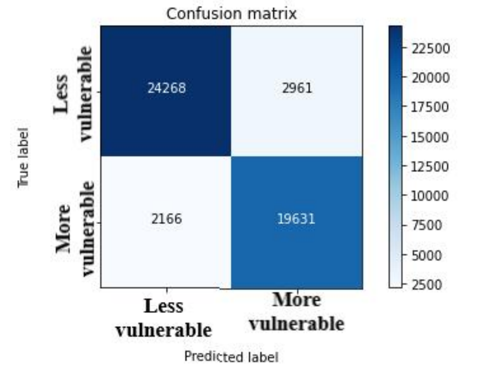	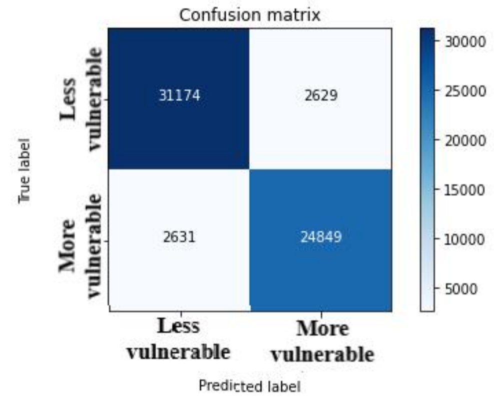	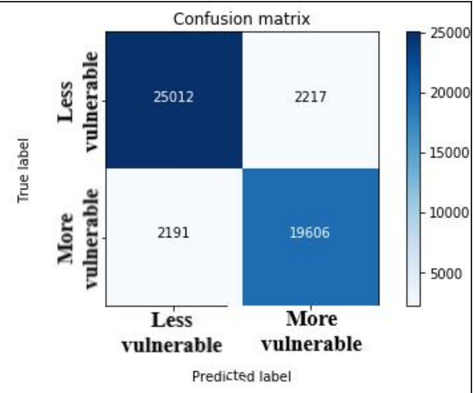
**Theta (4-8 Hz)**	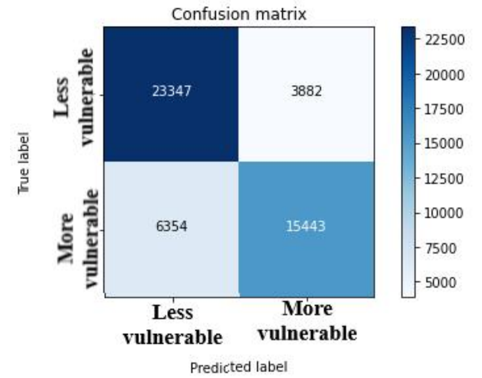	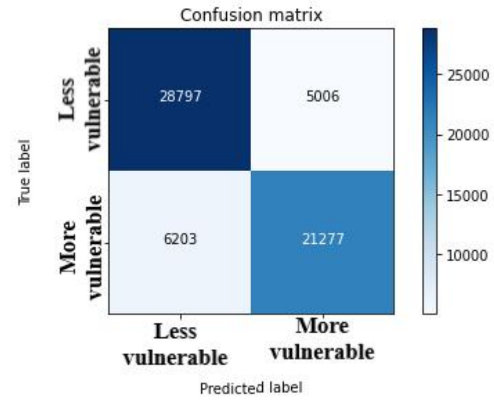	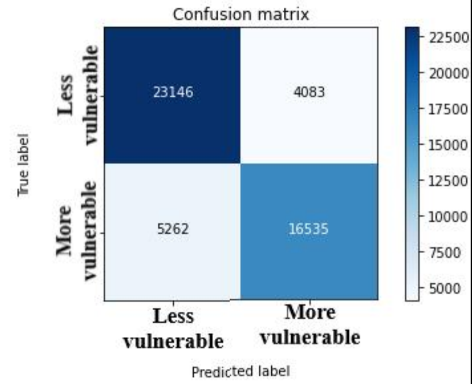
**Alpha (8-12 Hz)**	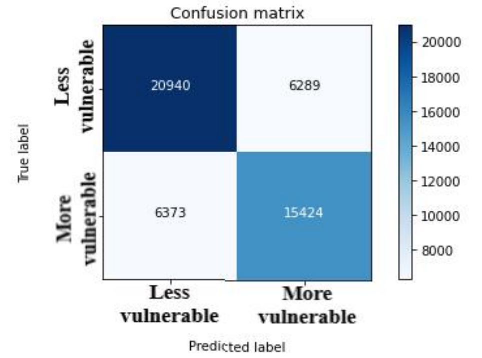	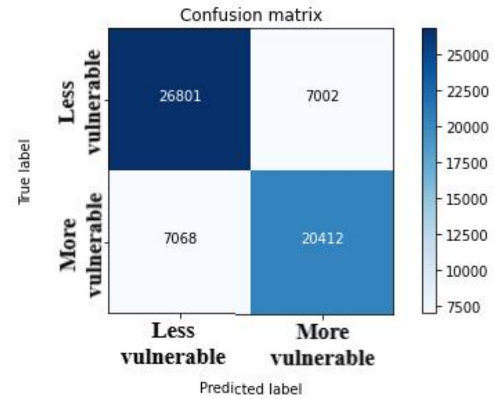	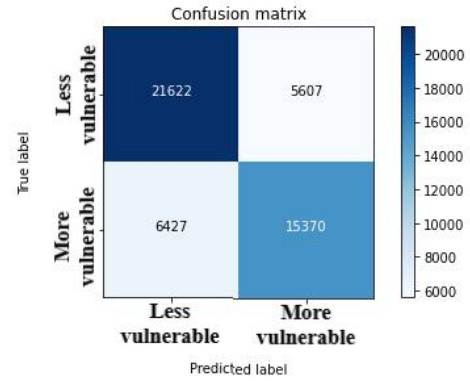
**Beta (12-28 Hz)**	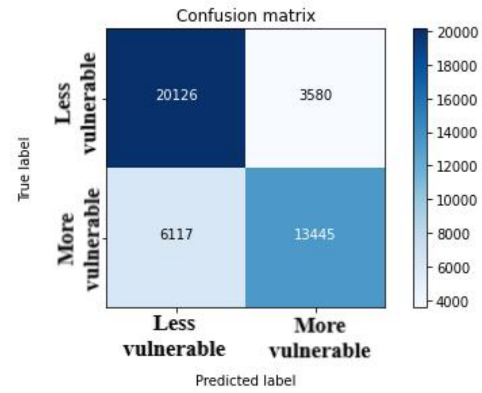	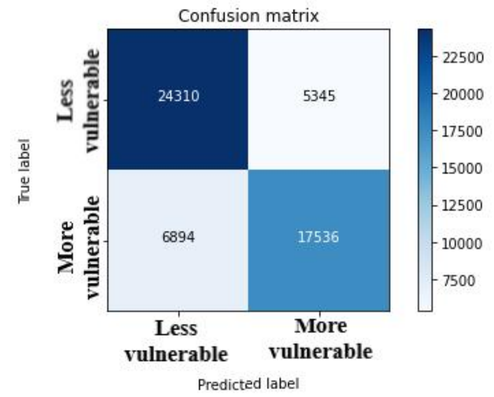	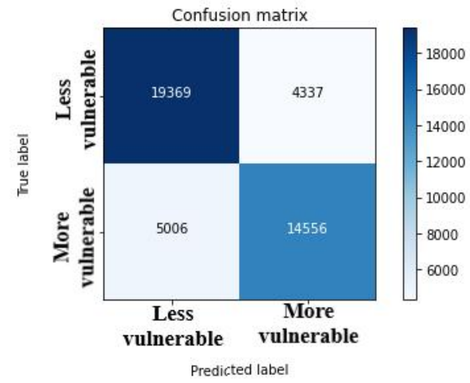

**Gamma (28-40 Hz)**	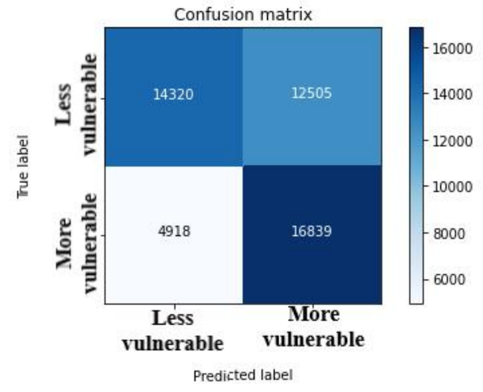	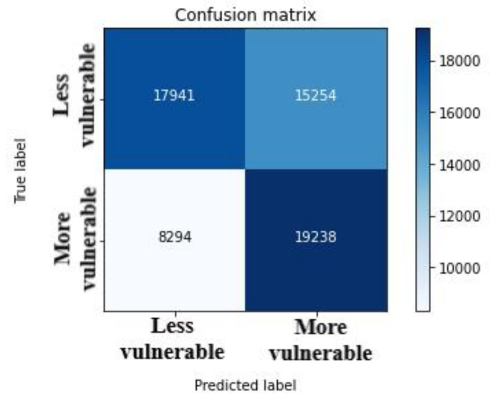	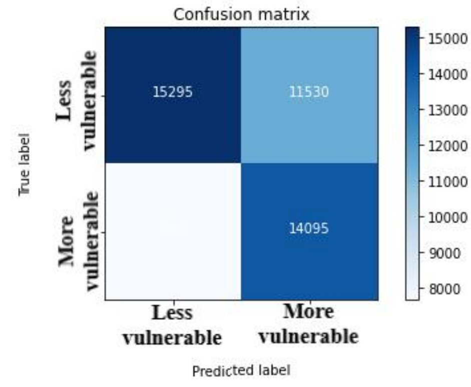

Supplement Table VIII. The confusion matrices for classification results of **task-based EEG data** using MultiLayer Perceptron (MLP) and Decision Tree (DT).

	**MLP**	**DT**
**Raw**	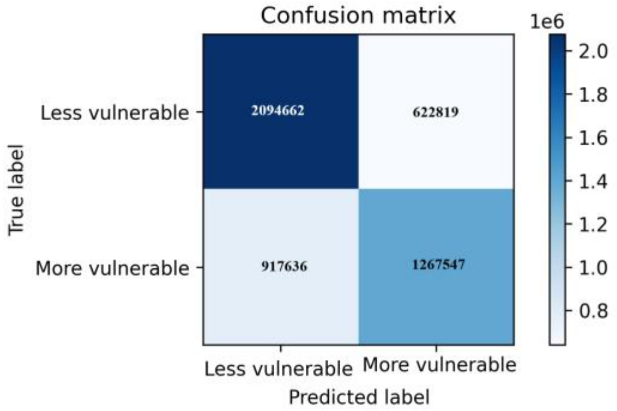	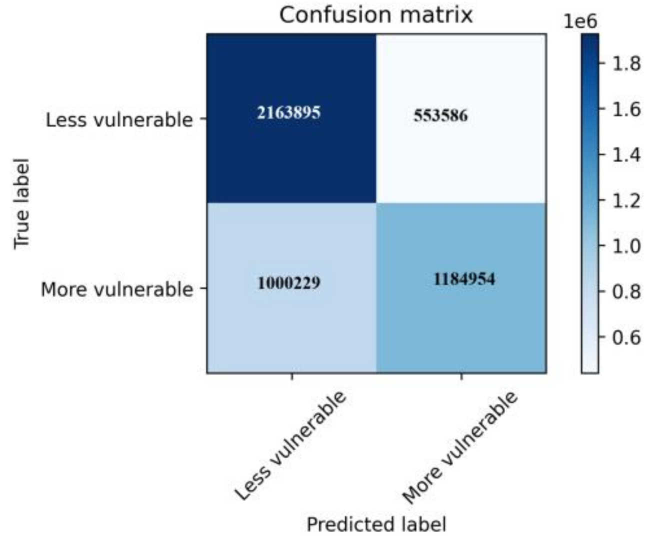
**Delta (0.5-4 Hz)**	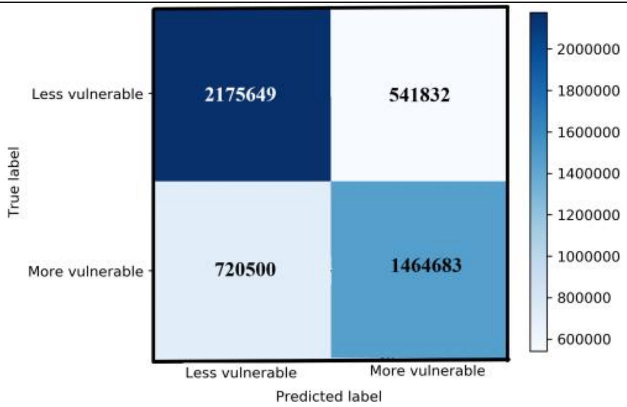	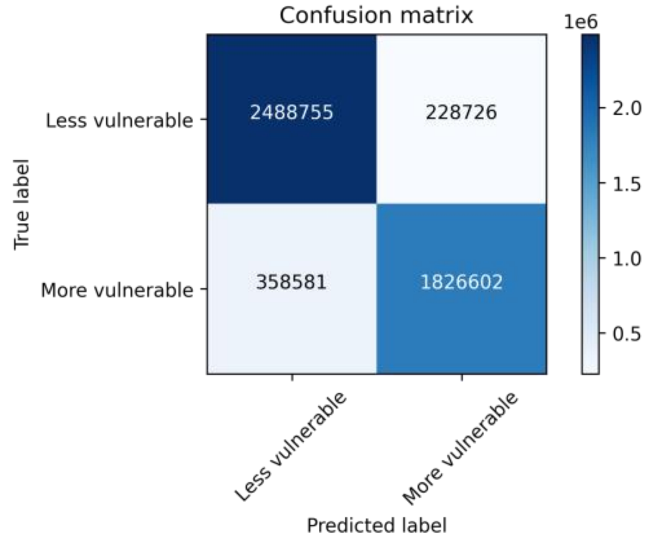

<table>
<tr>
<td>Theta (4-8 Hz)</td>
<td>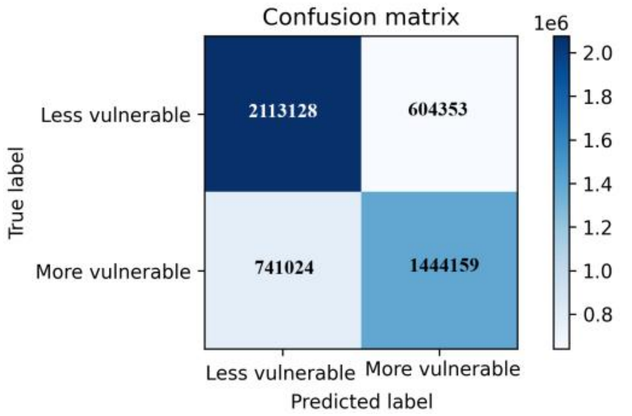
</td>
<td>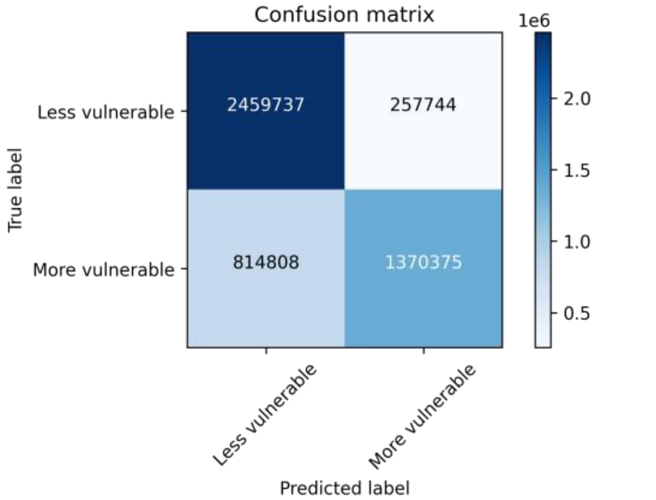
</td>
</tr>
<tr>
<td>Alpha (8-12 Hz)</td>
<td>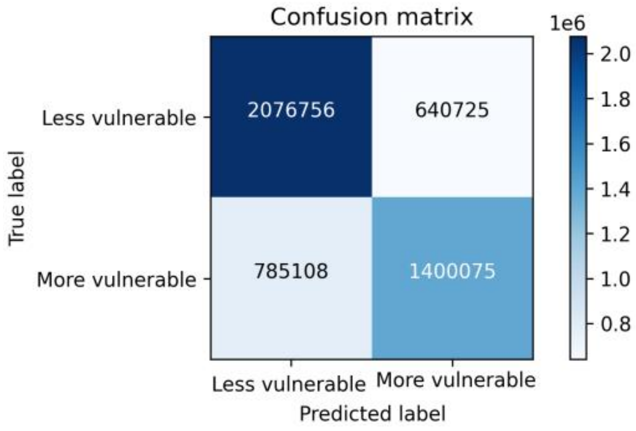
</td>
<td>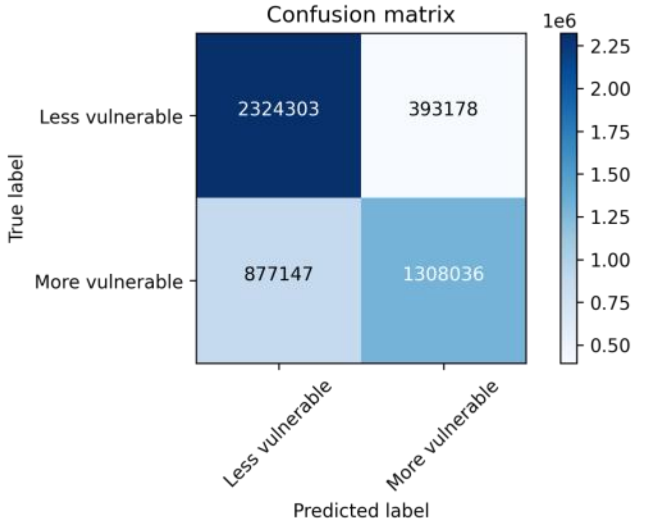
</td>
</tr>
<tr>
<td>Beta (12-28 Hz)</td>
<td>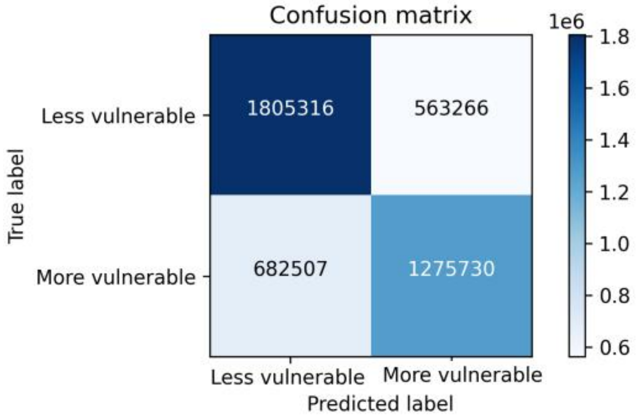
</td>
<td>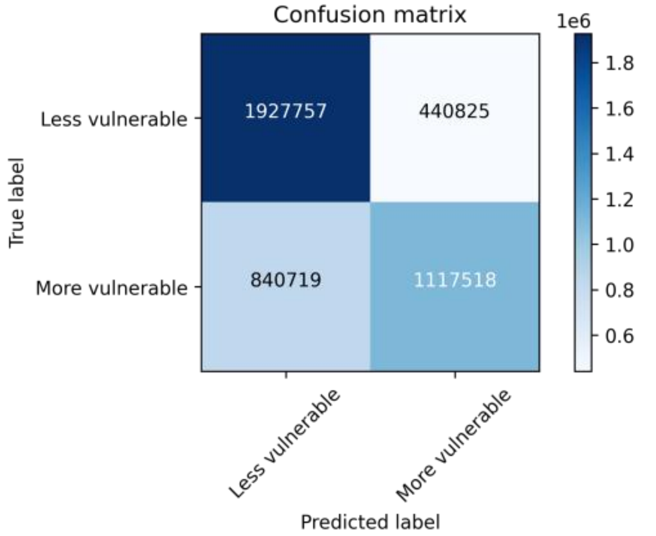
</td>
</tr>
</table>

<table>
<tr><td>Gamma(28-40 Hz)</td><td>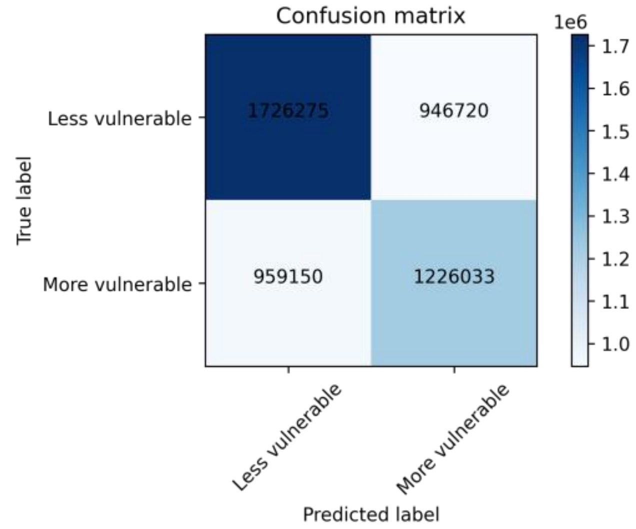
</td><td>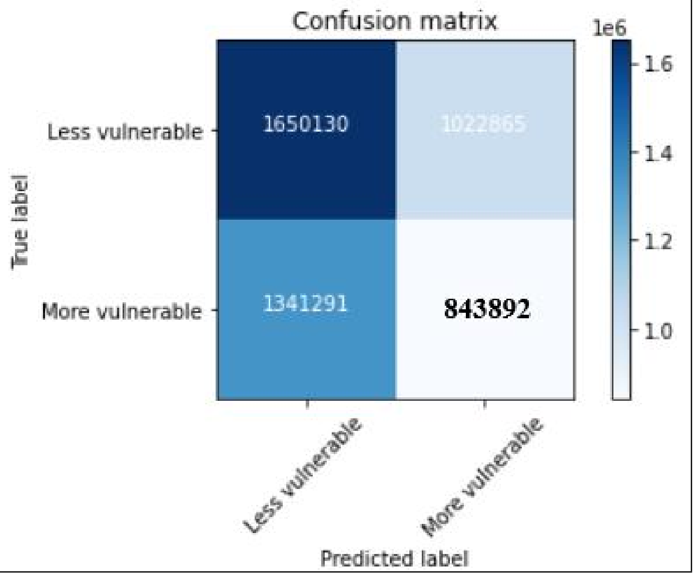
</td></tr>
</table>

Supplement Table IX. The confusion matrices for classification results of **<u>rs-EEG</u>** using MultiLayer Perceptron (MLP) and Decision Tree (DT).

<table>
<tr><td></td><td>MLP</td><td>DT</td></tr>
<tr><td>Raw</td><td>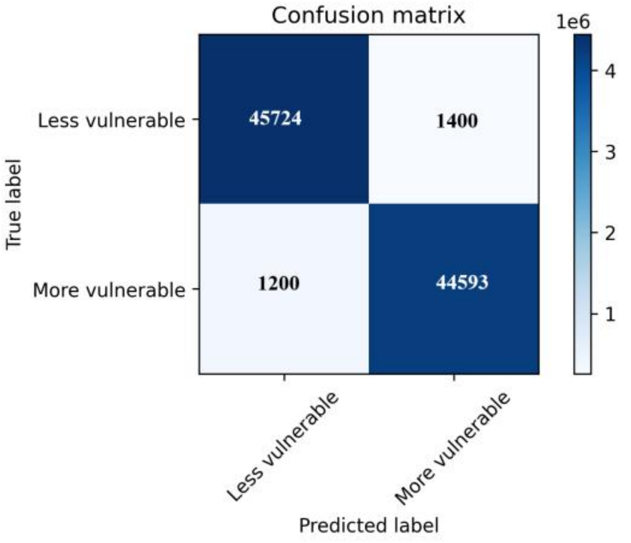
</td><td>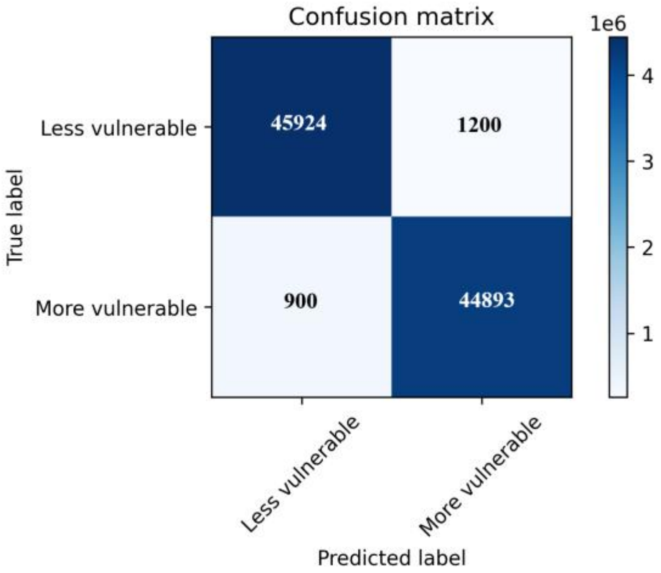
</td></tr>
</table>

Delta (0.5-4 Hz)

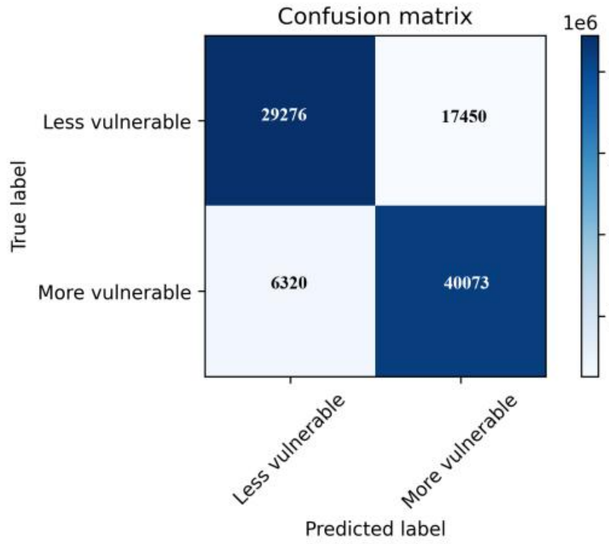

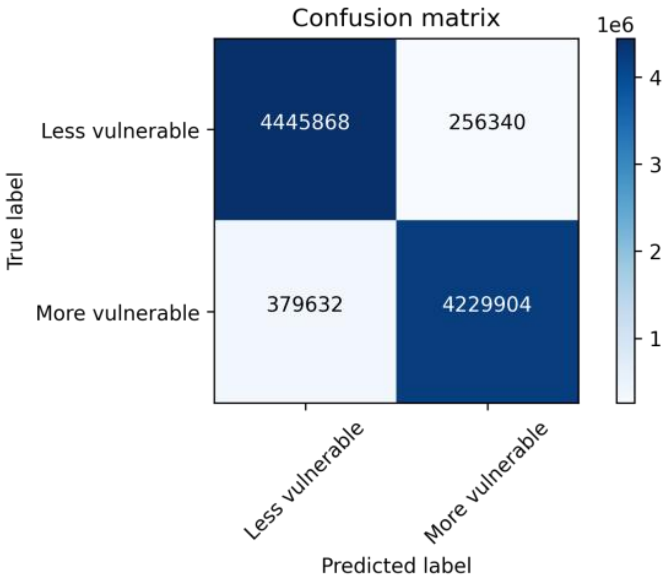

Theta (4-8 Hz)

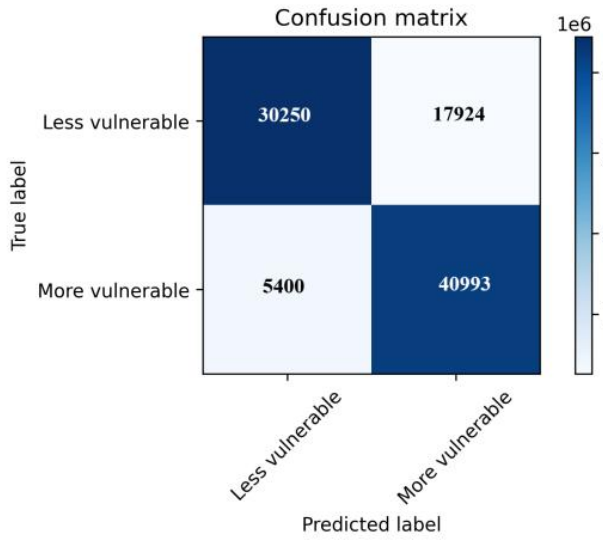

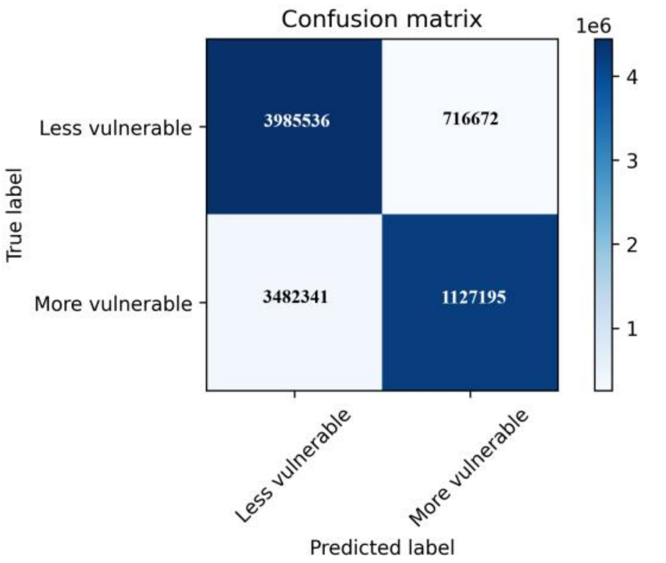

Alpha (8-12 Hz)

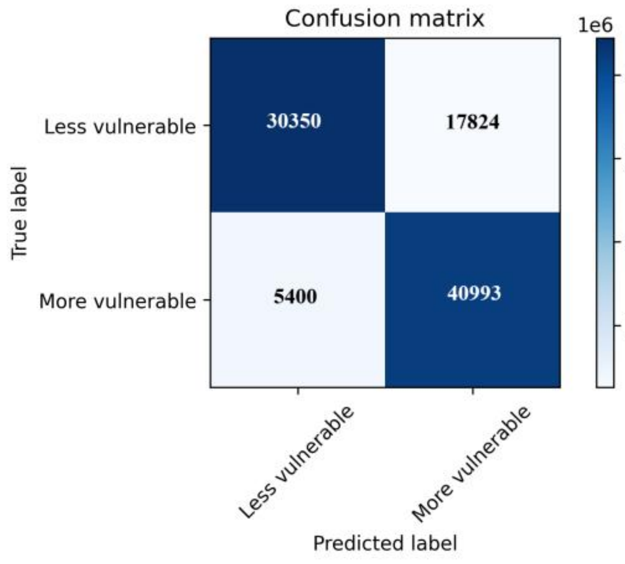

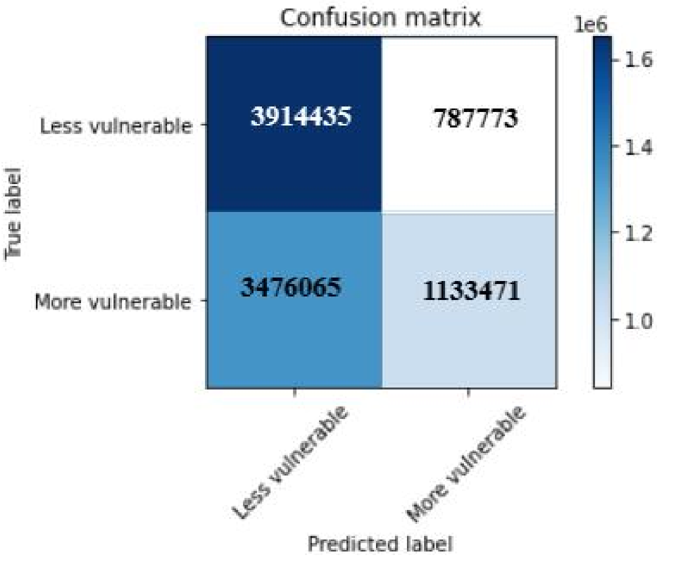

<table>
<tr>
<td>Beta (12-28 Hz)</td>
<td>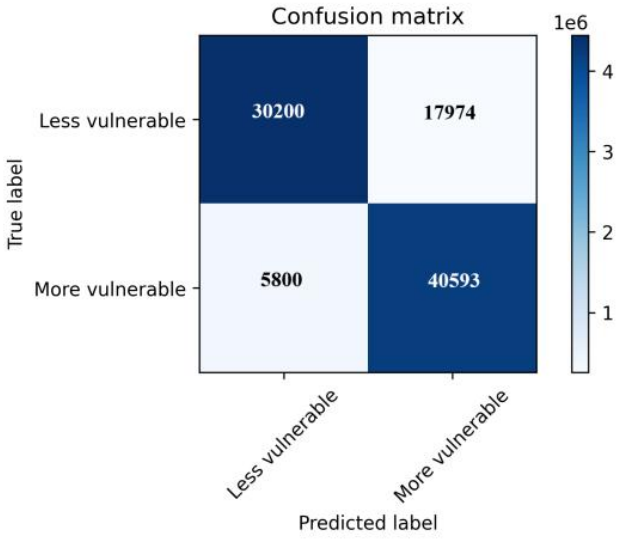
</td>
<td>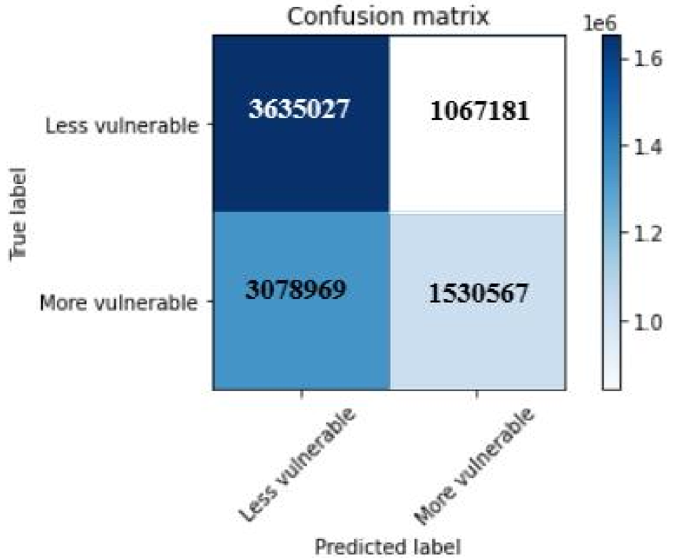
</td>
</tr>
<tr>
<td>Gamma (28-40 Hz)</td>
<td>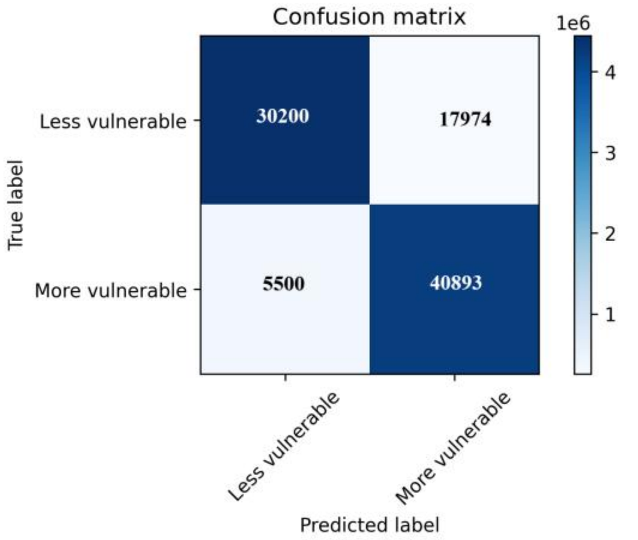
</td>
<td>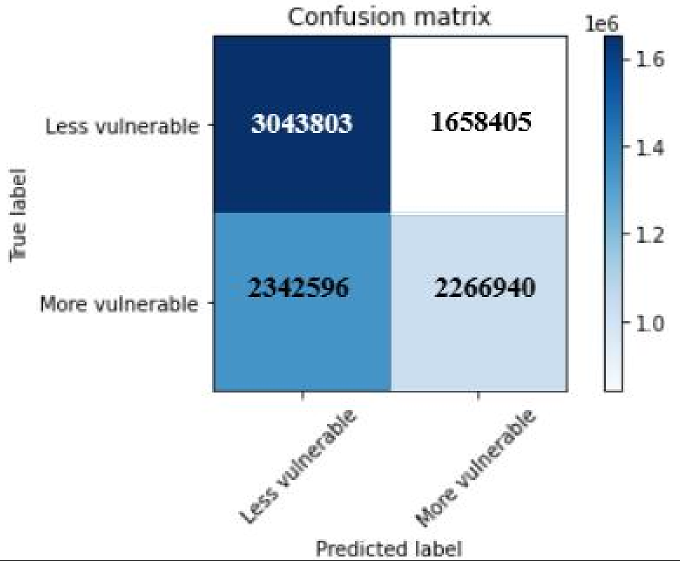
</td>
</tr>
</table>

References

[1] Rumelhart, D. E., Hinton, G. E. & Williams, R. J. Learning representations by back-propagating errors. nature 323, 533–536 (1986).

[2] Zhao, X. et al. Classification of sleep apnea based on EEG sub-band signal characteristics. Sci. Reports 11, 1–11 (2021).

[3] Emary, E., Zawbaa, H. M., & Hassanien, A. E. Binary grey wolf optimization approaches for feature selection. Neurocomputing, 172, 371-381 (2016).
